# A meta-analysis on the impact of concurrent or pre-existing cancer diagnosis on acute myocardial infarction outcomes

**DOI:** 10.1371/journal.pone.0318437

**Published:** 2025-01-31

**Authors:** Jie Wang, Jia Yu

**Affiliations:** 1 Department of Geriatric Internal Medicine, Huzhou Third Municipal Hospital, The Affiliated Hospital of Huzhou University, Huzhou, Zhejiang, China; 2 Department of Geriatric Psychiatry, Huzhou Third Municipal Hospital, The Affiliated Hospital of Huzhou University, Huzhou, Zhejiang, China; Sichuan University, CHINA

## Abstract

**Background:**

There is still a significant gap in understanding the impact of concomitant or previous cancer diagnoses on clinical outcomes of acute myocardial infarction (AMI)

**Objective:**

To provide updated evidence on the effect of concomitant or previous cancer diagnoses on mortality and risk of complications, specifically major bleeding, myocardial reinfarction, and stroke, of patients with AMI.

**Methods:**

A literature search was conducted across PubMed, EMBASE, and Scopus databases. English-language cohort studies published in peer-reviewed journals were included. Pooled effect estimates were calculated using random-effects models and reported as odds ratio (OR) or hazards ratio (HR) with 95% confidence intervals (CI). The certainty of the evidence was assessed using the standard GRADE approach.

**Results:**

A total of 22 studies were included. AMI patients with previous or concurrent cancer had increased risk of in-hospital mortality (OR 1.44, 95% CI: 1.20, 1.73), in-hospital mortality related to cardiovascular complications (OR 2.06, 95% CI: 1.17, 3.65), mortality at 30-days follow up (OR 1.47, 95% CI: 1.24, 1.74) and mortality at 1 year follow up (HR 2.67, 95% CI: 1.73, 4.11), compared to patients without cancer. The risk of major bleeding (OR 1.74, 95% CI: 1.40, 2.16), reinfarction (OR 1.20, 95% CI: 1.05, 1.37), and stroke (OR 1.16, 95% CI: 0.99, 1.37) was also higher in patients with previous or concurrent cancer. The certainty of evidence was rated as "low" for all outcomes, except for the risk of major bleeding, which was rated as "very low."

**Conclusion:**

Based on the low to very low certainty of evidence, we conclude that the presence of previous cancer diagnosis or concurrent cancer may increase the risk of adverse outcomes in patients with AMI. Early interventions, such as close monitoring of cardiac function, lifestyle modifications, and targeted pharmacological therapies, might help mitigate the risk of AMI and improve overall clinical outcomes. However, further methodologically rigorous studies are needed to validate the findings of this review.

## Introduction

Cardiovascular diseases, such as acute myocardial infarction (AMI), and cancer are among the most substantial contributors to global morbidity and mortality [1–3]. Advances in early detection and improvements in cancer treatments have led to an ever-increasing population of cancer survivors. However, such patients often face long-term health implications and are at a higher risk for cardiovascular incidents [4,5]. The increased risk of cardiovascular morbidities persists even after cancer treatments have been completed [6,7]. Furthermore, both cancer and AMI share risk factors such as old age, smoking, hypertension, diabetes, and obesity [8,9]. Malignancy exerts detrimental effects on the cardiovascular system. Chronic inflammation, a hallmark of cancer, can induce and exacerbate atherosclerosis, leading to an increased risk of cardiovascular diseases [10,11]. Additionally, cancer-associated factors can promote endothelial damage, impair vascular function, and disturb the delicate balance of thrombotic and fibrinolytic processes, further contributing to cardiovascular complications [12,13].

Due to the acute, life-threatening nature of AMI, understanding the impact of a cancer diagnosis on AMI outcomes is crucial for optimizing patient management and improving overall care. While there is a substantial body of literature examining the impact of pre-existing or concurrent cancer on AMI outcomes, this association is still unclear due to the lack of consistency across studies. Existing studies vary in their designs, populations, and reported outcomes, making it difficult to draw definitive conclusions about the overall risks that cancer patients face when experiencing AMI.

Previous meta-analyses attempted to address this issue but with certain limitations [14,15]. Dongchen et al. conducted a meta-analysis that incorporated data from 10 studies. However, their analysis missed some of the published studies on the topic [14]. The key findings of the meta-analysis were that cancer patients experiencing AMI had increased risks of all-cause mortality, recurrent myocardial infarction, and major bleeding. However, no notable disparities were observed in the risk of mortality attributable to cardiovascular causes or the risk of stroke. Similarly, another meta-analysis conducted by Balakrishna et al. only included 7 studies with percutaneous coronary intervention (PCI), which significantly limited the scope of the analysis [15], and found that cancer patients undergoing PCI had higher rates of mortality and need for blood transfusion. However, no significant variances in the risk of myocardial reinfarction or stroke were identified.

The current meta-analysis aimed to provide updated evidence on the impact of concomitant or previous cancer diagnoses on the clinical outcomes of patients with AMI. The outcomes of interest for this study are mortality and risk of complications (major bleeding, myocardial reinfarction and stroke).

## Methodology

### Compliance with relevant guidelines

PRISMA guidelines were followed [16] (S1 File). The study protocol was registered in PROSPERO (CRD42024526099).

### Identification of studies

Medline via PubMed, Embase, and Scopus databases were searched. The search terms included a combination of keywords: (active malignancy or concurrent cancer or cancer survivor or prior cancer) AND (cardiac disease or acute myocardial infarction OR myocardial infarction or AMI or myocardial ischemia) AND (mortality or survival or complications or clinical outcome or prognosis). The detailed search strategy for each of the three databases has been presented in S1–S3 Tables. The search was limited to studies published until 10th March 2024. Furthermore, to ensure thoroughness, we conducted manual searches of reference lists and relevant review articles, thus supplementing the electronic search process to include any additional studies that might have been overlooked.

The outcomes of interest were mortality and complications (major bleeding, reinfarction, and stroke). The operational definitions for major bleeding, reinfarction, and stroke were adopted as specified in the individual studies. For the mortality outcome, the primary interest was to examine risk of mortality within one year of follow-up, including in-hospital mortality and mortality within 30 days. Additionally, we also examined the risk of mortality at more than one year of follow-up.

Included studies focused on evaluating outcomes of interest in patients with both AMI and cancer (either previous or concurrent). A comparison group included patients with AMI but without a cancer diagnosis. We focused on studies reporting outcomes in the general population of patients with acute myocardial infarction (AMI). Studies that exclusively examined specific subgroups of AMI, such as patients with cardiogenic shock or cardiac arrest, were excluded. We preferred to include studies with a cohort design, either prospective or retrospective, and those that either presented confounder adjusted estimates or had done propensity score matching to account for baseline differences in participant characteristics. Only studies published in peer-reviewed journals, conducted on human subjects, and available in English were considered. Only studies that obtained a quality assessment score of 6 or more, from the maximum score of 9, based on the Newcastle-Ottawa Scale (NOS) assessment, were eligible for inclusion [17]. Studies not directly addressing the impact of cancer on AMI outcomes, case reports, letters, reviews, and conference abstracts, studies lacking a control group, studies with insufficient data, presenting unadjusted effect sizes or substantial bias were excluded.

After implementing the search strategy in the designated databases, studies were deduplicated. Subsequently, two authors reviewed the titles and abstracts of the remaining studies to assess their potential relevance to the research question. Selected studies showing potential relevance underwent further evaluation. In the subsequent stage, a thorough examination of the full text was conducted to ascertain the eligibility for inclusion. Any discrepancies or disagreements regarding study inclusion were resolved through discussions.

### Data extraction and quality assessment

The data extraction process from the final set of studies was independently conducted by two authors using a standardized data extraction form. In cases of any disparities between the two authors, discussions were initiated to reconcile and reach a consensus. The Newcastle-Ottawa Scale (NOS) was used to evaluate the risk of bias within the included studies [17]. Two study authors independently carried out the quality assessment.

### Statistical analysis

The pooled effect sizes were reported as odds ratios (OR) or hazard ratios (HR) with 95% confidence intervals (CI). A random-effects model was used for all the analyses to account for the differences in the baseline characteristics of the studies [18]. Egger’s test and funnel plots were used to assess publication bias [19]. A p-value less than 0.05 denoted statistical significance. Subgroup analyses were conducted based on whether the study included subjects with prior or concurrent cancer and whether percutaneous intervention (PCI) was the primary treatment modality. We evaluated the certainty of the evidence using the standard GRADE approach (gradepro.org) and GRADE Pro Software [20].

## Results

**Fig 1:**
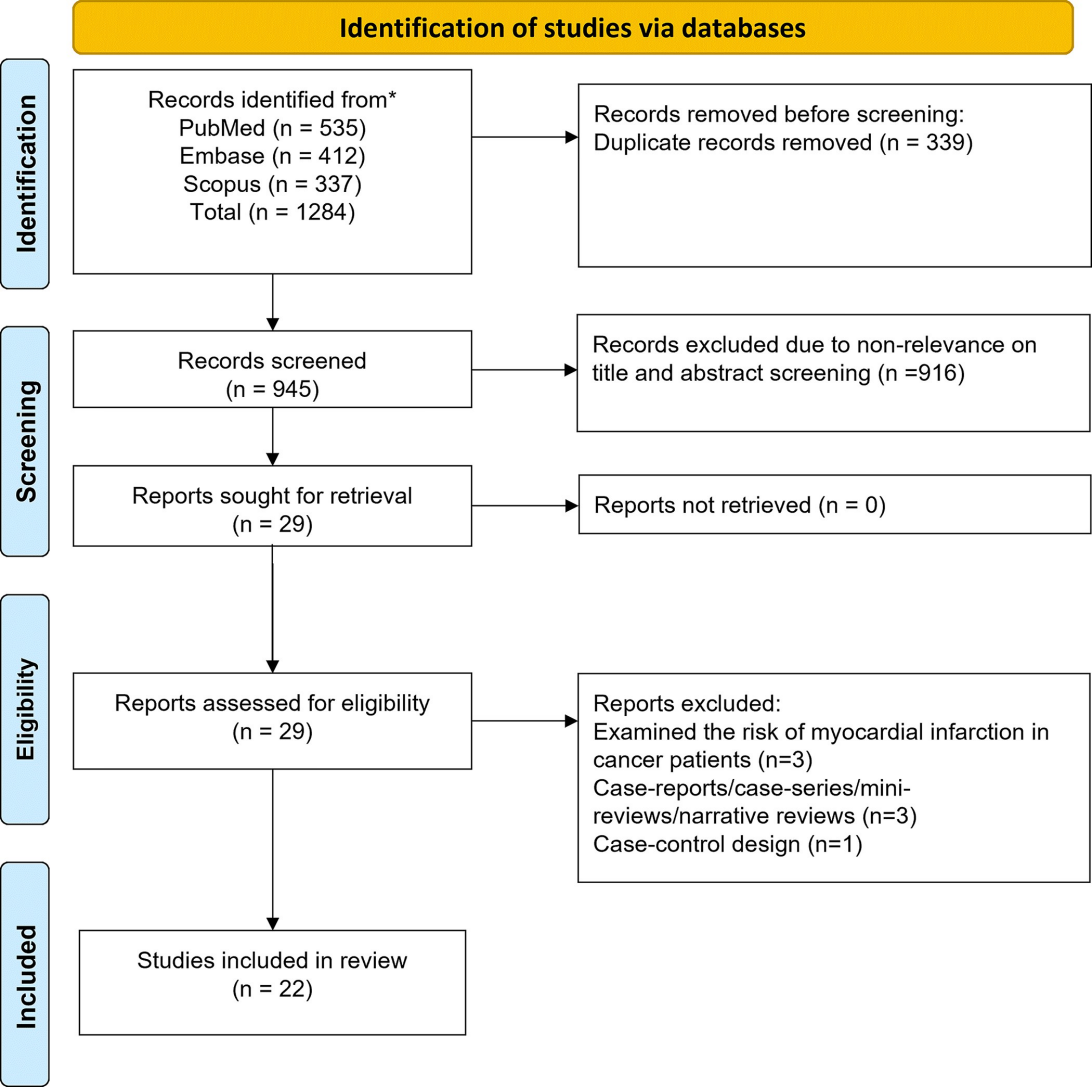
Study selection process.

The initial search across the databases yielded 1284 studies. After eliminating 339 duplicates, the titles and abstracts of the remaining 945 studies underwent screening, and an additional 916 studies were excluded. A thorough full-text review of the remaining 29 studies was conducted, excluding 7 additional studies that did not meet our criteria (S2 File). The screening process and the reasons for exclusion are described in Fig 1. No studies were excluded solely based on obtaining an NOS score of less than 6. Our final analysis comprised 22 studies [21–42]. Detailed study information is provided in Table 1.

**Table 1:** Characteristics of the included studies.

Author (publication year)	Place of study and study design	Type of AMI; PCI as a treatment modality	Age and sex distribution	Co-morbidity status	Sample size (n)	Newcastle Ottawa quality (NOS) score
Dafaalla (2023) [21]	UK Retrospective (registry based)	All had ST-segment elevation MI (STEMI); PCI in majority (~60%)	Those with concomitant cancer were older [median age 75 vs. 66 years] and with a lower proportion of females.	Those with concomitant cancer had a higher prevalence of chronic diseases and co-morbidities	With concomitant cancer (8581) Without cancer (3,44,867)	9
Hayashi (2022) [22]	Japan Retrospective	STEMI; (~80%) All patients received PCI	Patients with prior cancer were older (mean age 77 vs. 68 years) and mostly females	Those with prior cancer had a higher prevalence of co-morbidities	With prior cancer (79) Without cancer (633)	8
Koo (2022) [23]	Singapore Retrospective	STEMI (~56%); Data not provided	Patients with prior cancer were older (median age of 73 years vs. 61 years) with more females	Those with prior cancer had a higher prevalence of hypertension	With prior cancer (1086) Without cancer (17,114)	7
Nozaka (2020) [24]	Japan Retrospective	Not provided; Majority (>85%) of subjects received PCI	Patients with previous cancer were older than those without cancer (mean age of 74 years vs. 66 years).	There were no significant differences in co-morbidities	With prior cancer (50) Without cancer (1245)	7
Takeuchi (2023) [25]	Japan Retrospective	STEMI (~72%); Majority (>95%) of subjects received PCI.	Those with prior cancer were older (mean age of 77 years vs. 66 years)	The proportion of patients with co-morbidities similar in both groups.	With prior cancer (55) Without cancer (496)	7
Ye_A (2023) [26]	United States Retrospective (Medical Information Mart for Intensive Care IV (MIMIC-IV) database	Not reported; Majority did not receive PCI (60%)	Patients were older in the cancer group (73 yrs vs. 70 yrs), with a similar sex distribution.	Comorbidities were higher in the cancer group.	With concomitant cancer (297) Without cancer (2737)	8
Ye_B (2023) [26]	United States Retrospective (eICU Collaborative Research Database (eICU-CRD))	Not reported; Majority did not receive PCI (>75%)	Patients were older in the cancer group (75 yrs vs. 65 yrs) with a lower proportion of males (58% vs. 63%).	Comorbidities were higher in the cancer group.	With concomitant cancer (578) Without cancer (5390)	8
Velders (2013) [27]	Netherlands Retrospective	All patients with STEMI; All patients underwent PCI	Patients with cancer were older (mean age of 70 years vs. 63 years), with a higher proportion of females (32% vs. 24%)	Comorbidities were higher in the cancer group.	With prior cancer (208) Without cancer (3215)	7
Wang (2016) [28]	United States Retrospective	All patients with STEMI; All patients underwent PCI	Those with cancer were older (mean age of 72 years vs. 63 years), with a higher proportion of females (32% vs. 29%)	Comorbidities were higher in the cancer group.	With prior cancer (261) Without cancer (1313)	9
Landes (2017) [29]	Israel Retrospective	All patients with STEMI; All patients underwent PCI	Similar age (around 77 years) in both groups; higher proportion of females in those with previous cancer (78% vs. 28%)	A higher proportion in the previous cancer group had experienced a stroke and had previous anti-coagulation therapy	With prior cancer (969) Without cancer (969)	8
Iannaccone (2018) [30]	Multicentric in Europe Retrospective	All patients with STEMI; All patients underwent PCI	Those with cancer were older (mean age 71 years vs. 63 years), with a higher proportion of females (29% vs. 23%).	Comorbidities were higher in the cancer group	With concomitant cancer (858) Without cancer (13773)	8
Nakatsuma (2018) [31]	Japan Retrospective	Not provided; All patients underwent PCI	Those with cancer were older (mean age 73 years vs. 68 years), with a similar sex distribution	There were no significant differences in co-morbidities	With prior cancer (1109) Without cancer (11071)	8
Potts (2020) [32]	United States Retrospective	Similar proportion with STEMI (~22%) and Non-STEMI (~25%) All patients underwent PCI	Those with cancer were older (mean age 73 years vs. 65 years) and had a lower proportion of females (27% vs. 34%)	Similar proportion of patients with co-morbidities in both groups.	With concomitant cancer (15789) Without cancer (6,545,656)	7
Guo (2021) [33]	United States Retrospective	Similar proportion with STEMI (~20%) and Non-STEMI (~26%); All patients underwent PCI.	There was a similar age distribution (mean age 72 years) and sex distribution (33% females)	A higher proportion of patients with diabetes and hypertension in cancer group	With concomitant cancer (416) Without cancer (768)	7
Peng (2022) [34]	China Retrospective	Majority with STEMI (65%); Most patients underwent PCI (~65%).	Similar age and sex distribution in both groups (mean age of 70 yrs and around 60% males)	A higher proportion of patients with previous CHD/ MI in the cancer group	With concomitant cancer (150) Without cancer (542)	8
Tosaka (2021) [35]	Japan Retrospective	Majority with STEMI (55%); Most patients underwent PCI (80%).	Those with active cancer were older (68 vs. 75 years) than those without cancer	Those with active cancer had a higher prevalence of renal deficiency, and a lower ejection fraction	With concomitant cancer (63) Without cancer (1027)	8
Zheng (2021) [36]	Japan Retrospective	Not provided; Most patients underwent PCI (>90%)	Those with cancer were older (73 years vs 68 years)) than those without cancer	There was a higher proportion of patients in the non-cancer group who had hypertension or dyslipidaemia.	With concomitant cancer (6995) Without cancer (6995)	7
Bharadwaj (2021) [37]	United States Retrospective	Around 30% with STEMI, no data provided about non-STEMI; PCI conducted in 44% of the patients.	Patients with cancer were older (median age of 75) with a proportion of females of 35%.	The proportion of patients with co-morbidities was higher in the cancer group.	With concomitant cancer (186604) Without cancer (5966955)	9
Ederhy (2019) [38]	France Retrospective	STEMI (~50%); Majority with PCI (~65%)	Patients with a history of cancer were older (mean age of 74 years)	Those with prior cancer had higher proportion with comorbidities	With prior cancer (246) Without cancer (3418)	8
Zadok (2019) [39]	Israel Retrospective	Non-STEMI (60%); Most patients underwent PCI (75%).	Patients with cancer were older (75 vs. 63 years) and more often females.	There was a higher proportion of patients with hypertension among those with cancer; similar proportion with diabetes in both groups	With concomitant cancer (152) Without cancer (2937)	8
Gong (2018) [40]	Canada Retrospective	Not provided; Most patients underwent PCI (>90%).	There was a higher proportion of men in both groups (around 60%) and those with prior cancer were older (>65 years)	Those with prior cancer had higher proportion with associated comorbidities	With prior cancer (22907) Without cancer (247182)	7
Kurisu (2012) [41]	Japan Retrospective	Not provided; PCI was conducted in ~20% of patients.	The mean age was similar in the two group (around 70 years), with a similar sex distribution (men 70%)	Similar distribution of comorbidities such as DM and hypertension	With concomitant cancer (18) Without cancer (59)	7
Velders (2020) [42]	Switzerland Retrospective	Not provided; Most patients underwent PCI (>90%).	Patients with cancer were older (around 70 years) with proportionately more women	Those with cancer had higher prevalence of co-morbidities	With prior cancer (208) Without cancer (3215)	8

NOS quality assessment scores of the included studies ranged from 7 to 9, with a mean score of 7.7, indicating high quality. The quality assessment of the individual studies is presented in S4 and S5 Tables. All included studies were retrospective; most used registry-based data or data from medical records. Most of the studies were conducted in Japan (n = 7), followed by five studies in the United States and two in Israel. One study each was conducted in France, the United Kingdom, Singapore, the Netherlands, China, Canada, and Switzerland. One multicentric study was conducted in different institutions across Europe. In 11 studies, the included patients had prior cancer and were cancer survivors. In the remaining 11 studies, the patients had concurrent cancer. In the majority of the studies (n = 16), the primary modality of management was PCI. There was one study by Ye et al (2023) that presented separate findings from the analysis of two different large databases (eICU Collaborative Research Database (eICU-CRD) and Medical Information Mart for Intensive Care IV (MIMIC-IV) database) [26]. Therefore, for the present analysis, this study was considered as two individual studies and labeled as Ye_A (2023) and Ye_B (2023) (Table 1). Most studies reported baseline differences among the study participants between the two groups (patients with or without previous/concurrent cancer) regarding age, sex distribution, and the proportion of patients with comorbidities. Patients with previous/concurrent cancer were usually older, of the male gender, and had a higher prevalence of diabetes, hypertension, peripheral vascular disease, previous stroke, and previous myocardial infarction (MI). The included studies had variability in the definitions adopted for non-mortality outcomes (major bleeding, reinfarction, and stroke). Most included studies reported confounder-adjusted findings or findings from multivariable regression analysis. Some studies employed propensity score matching to adjust baseline participant characteristics. In these cases, most characteristics were balanced between the groups, eliminating the need for further adjustment in the analytic model. The generation of pooled estimates in this meta-analysis was based on adjusted findings from each of the included studies.

### Mortality outcomes

**Fig 2:**
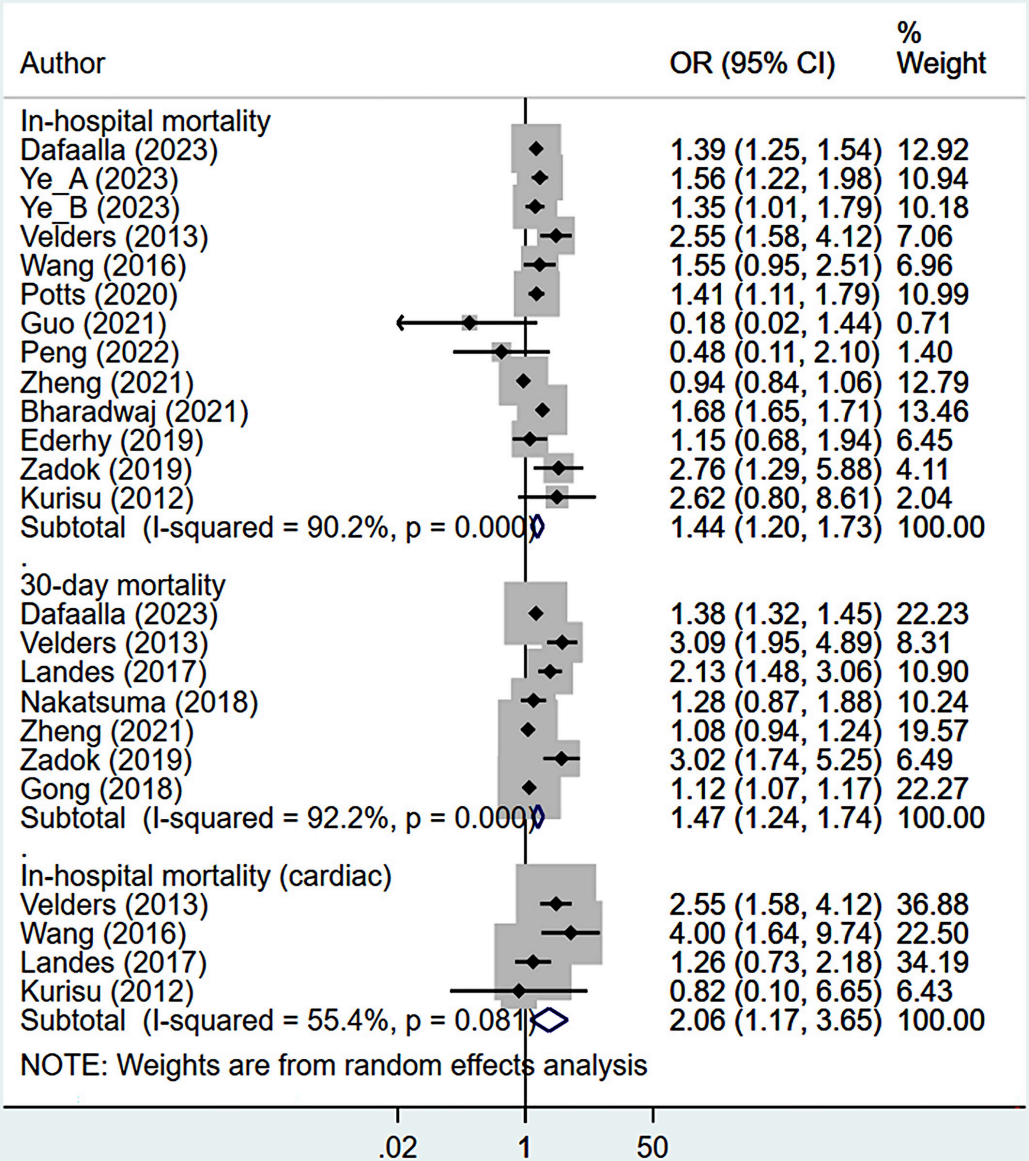
Pooled risk of in-hospital mortality (overall and due to cardiovascular complications) and 30-day mortality (overall).

**Fig 3:**
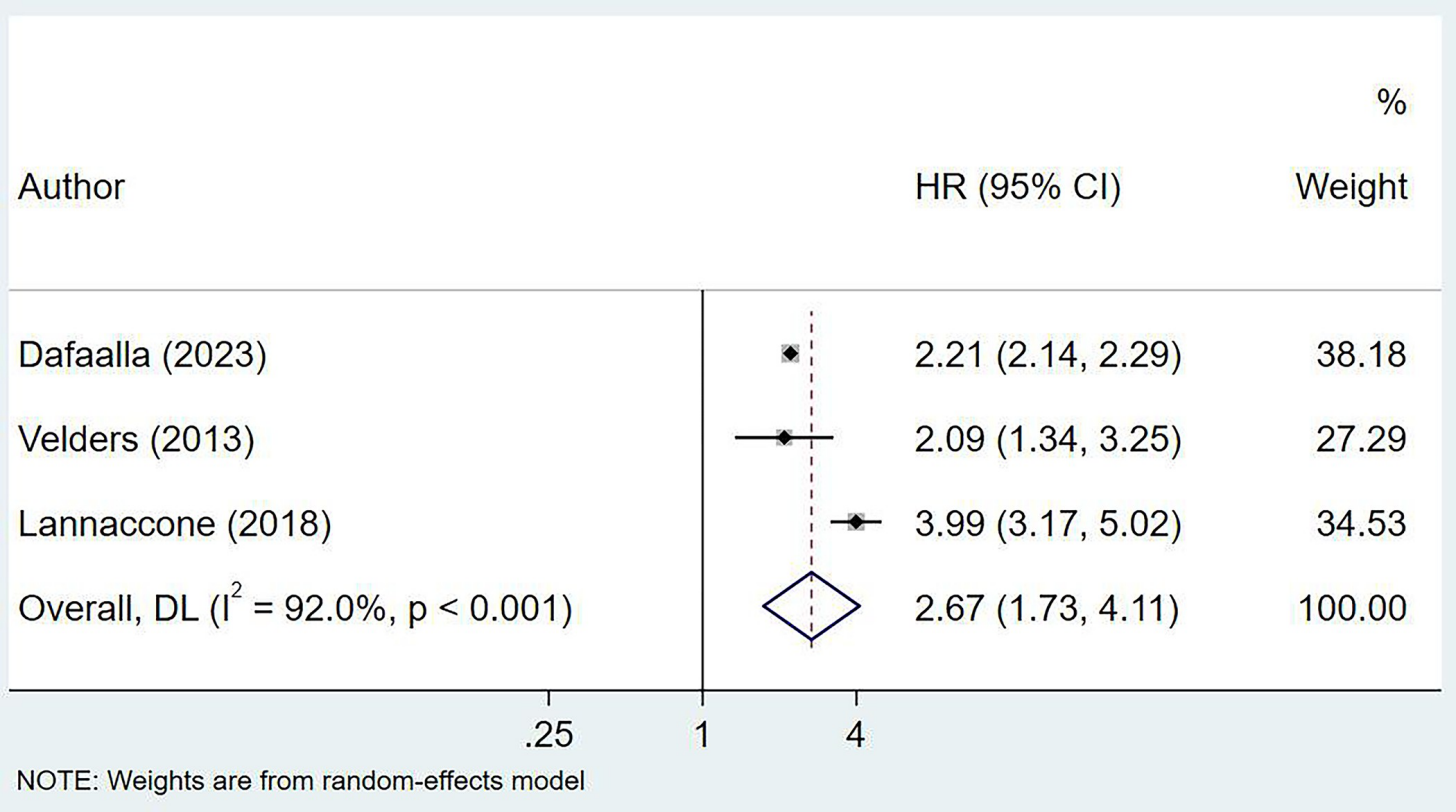
Pooled risk of mortality at 12 months follow up.

Patients with previous or concurrent cancer and AMI had an increased risk of in-hospital mortality (OR 1.44, 95% CI: 1.20, 1.73; n = 13, I^2^ = 90.2%), mortality at 30-days follow up (OR 1.47, 95% CI: 1.24, 1.74; n = 7, I^2^ = 92.2%) and mortality at 12 months of follow up (HR 2.67, 95% CI: 1.73, 4.11; n = 3, I^2^ = 92.0%) (Figs 2 and 3). AMI patients with previous or concurrent cancer exhibited a higher risk of in-hospital mortality related to cardiovascular complications (OR 2.06, 95% CI: 1.17, 3.65; n = 4, I^2^ = 55.4%) (Fig 2).

Despite the limitations of pooling findings from follow-up periods of more than one year, with varying durations, the analysis showed an increased risk of all-cause mortality in patients with previous or concurrent cancer (HR 1.87, 95% CI: 1.65, 2.11; n = 15, I^2^ = 94.0%), while no statistically significant increase in cardiovascular-related mortality was observed (HR 1.20, 95% CI: 0.96, 1.50; n = 10, I^2^ = 74.4%) for the same population (S1 Fig).

The examination of the funnel plots and Egger’s test (p>0.05) did not indicate the presence of publication bias for mortality outcomes at any of the above-mentioned time points (S2–S4 Figs). According to the GRADE assessment criteria, the overall certainty of evidence for mortality-related outcomes was “low” (S6 Table).

The subgroup analysis revealed that individuals with both prior and concurrent cancer had an elevated risk of mortality across all time points, including in-hospital, at 30-days follow-up, and at 1 year of follow-up (Table 2; S5–S13 Figs). When the analysis was restricted to studies with PCI as the primary intervention modality, the risk of mortality continued to be higher at all time points in patients with previous or concurrent cancer compared with patients who did not have a cancer diagnosis (Table 2; S5–S13 Figs).

**Table 2:** Subgroup analysis.

	In-hospital mortality	30-day mortality	Mortality at 1 year follow up	Major bleeding	Reinfarction	Stroke
	**Effect size (95% CI) (n, I** ^**2**^ ) *					
**PCI**	1.59 (1.17, 2.15) (9; 84.6%)	1.60 (1.24, 2.07) (6; 88.4%)	2.97 (1.58, 5.59) (2; 84.5%)	1.68 (1.41, 2.00) (9; 75.5%)	1.28 (1.10, 1.49) (10; 90.4%)	1.08 (0.98, 1.19) (9; 40.4%)
**Prior**	1.67 (1.06, 2.63) (3; 60.2%)	1.70 (1.07, 2.70) (4; 90.1%)	2.09 (1.34, 3.25) (1; —)	1.45 (1.25, 1.69) (5; 43.3%)	1.03 (0.93, 1.14) (7; 76.4%)	1.10 (0.94, 1.29) (6; 52.7%)
**Concomitant**	1.39 (1.12, 1.71) (10; 92.3%)	1.42 (1.08, 1.87) (3; 89.5%)	2.94 (1.65, 5.24) (2; 96.0%)	1.85 (1.43, 2.38) (7; 92.0%)	1.48 (0.85, 2.56) (5; 93.4%)	1.20 (0.93, 1.54) (5; 91.7%)

*Effect size reported as odds ratio (OR) for all outcomes except mortality at 1 year follow up where the effect size is reported as Hazard ratio (HR)

### Risk of complications

**Fig 4:**
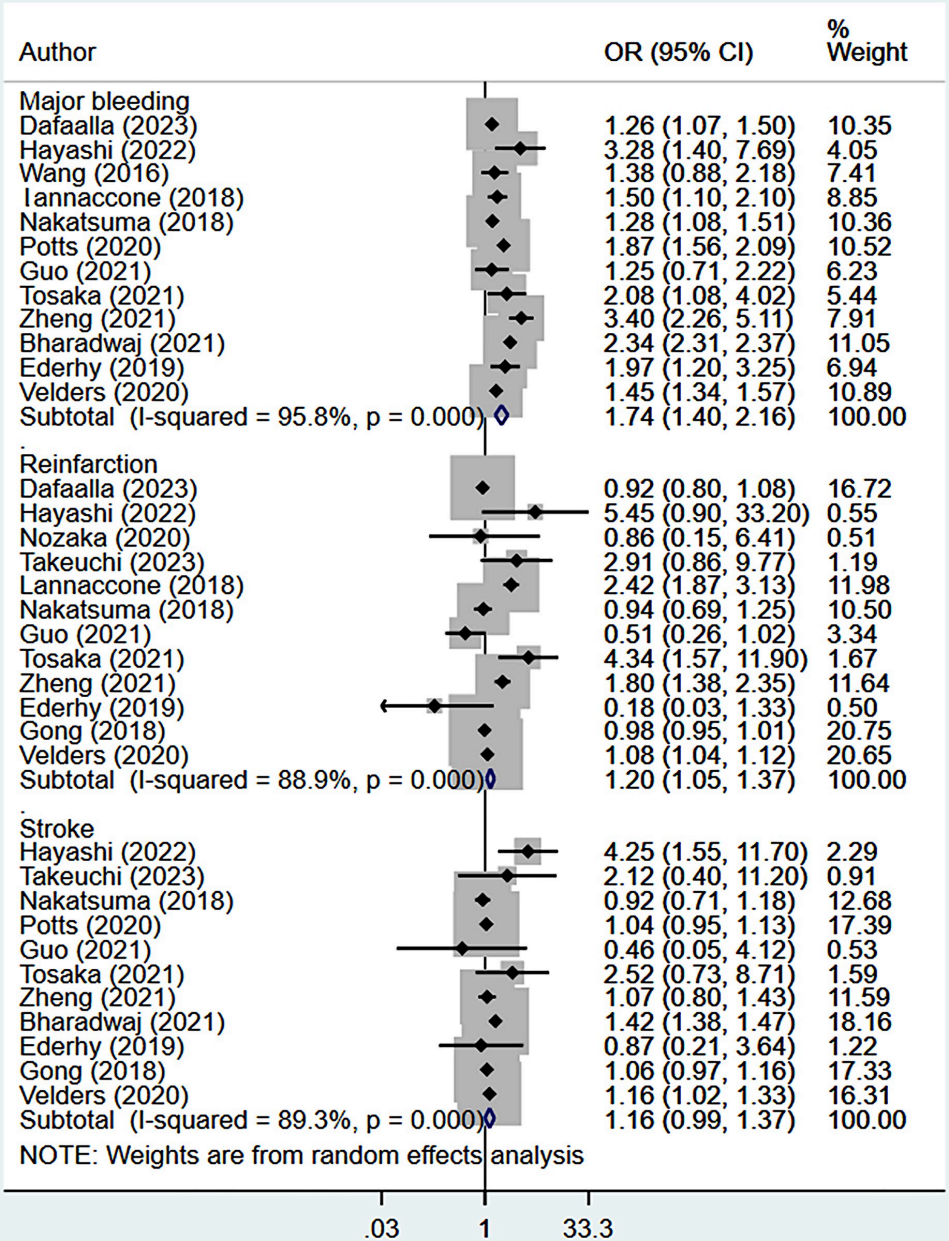
Pooled risk of complications.

In patients with previous or concurrent cancer, the risk of major bleeding (OR 1.74, 95% CI: 1.40, 2.16; n = 12, I^2^ = 95.8%) and reinfarction (OR 1.20, 95% CI: 1.05, 1.37; n = 12, I^2^ = 88.9%) was significantly higher compared to patients without cancer (Fig 4).

Patients with AMI and previous or concurrent cancer were at a higher risk of stroke (OR 1.16, 95% CI: 0.99, 1.37; n = 11, I^2^ = 89.3%. However, the difference was not statistically significant (Fig 4). The presence of publication bias for the outcome of "major bleeding" was detected, as indicated by both the funnel plot and Egger’s test (p = 0.04) (S14 Fig). However, no indication of publication bias was found for the outcomes of reinfarction and stroke based on the same assessments (S15 and S16 Figs). The certainty of evidence for the risk of major bleeding was assessed as "very low," while the certainty for the risk of reinfarction and stroke was rated as "low," based on the GRADE assessment criteria (S6 Table).

Subgroup analyses showed that the risk of major bleeding was increased in patients with previous cancer (OR 1.45, 95% CI: 1.25, 1.69; n = 5, I2 = 43.3%), concurrent cancer (OR 1.85, 95% CI: 1.43, 2.38; n = 7, I^2^ = 92.0%) and patients managed using PCI (OR 1.68, 95% CI: 1.41, 2.00; N = n = 9, I^2^ = 75.5%) (Table 2; S5–S13 Figs). The risk of reinfarction was significantly higher in patients undergoing PCI (OR 1.28, 95% CI:1.10, 1.49; n = 10; I^2^ = 90.4%) (S20 Fig). While not statistically significant, patients with concurrent cancer (but not prior cancer) had a higher risk of reinfarction compared to those without cancer (Table 2; S21 and S22 Figs). No statistically significant difference in the risk of stroke was detected by subgroup analyses based on the PCI as a management modality, prior cancer, or concurrent cancer (Table 2; S23–S25 Figs).

## Discussion

The present meta-analysis aimed to assess the impact of previous or concurrent cancer diagnoses on the risk of mortality and other complications in patients with AMI. The findings of our meta-analysis revealed a significant association between previous or concurrent cancer diagnoses and increased mortality, risk of major bleeding, reinfarction, and possibly stroke in this group of patients. The certainty of the evidence for the pooled findings was “low” to “very low”. Therefore, further robust and methodologically rigorous studies are needed to confirm these conclusions.

Our findings are consistent with the outcomes of two previous meta-analyses on this subject [14,15]. More specifically, our findings further confirm the results reported by Dongchen et al wherein patients with AMI were found to have a higher risk of mortality (all-cause), recurrent myocardial infarction, and major bleeding [14]. This review noted no significant difference in the risk of mortality due to cardiovascular causes or the risk of stroke. Another meta-analysis conducted by Balakrishna et al also documented that cancer patients undergoing PCI had higher rates of mortality and a need for blood transfusion (indicating major bleeding) [15]. However, the review did not find significant differences in the risk of myocardial reinfarction and stroke.

The underlying mechanisms for increased risk of mortality could be multifactorial. Cancer is associated with chronic inflammation, which can promote atherosclerosis, impair vascular function, and increase the risk of thrombotic events [10,43,44]. Furthermore, cancer-related treatments such as chemotherapy, radiation, and targeted therapies may have cardiotoxic and vascular toxic effects, leading to cardiac and vascular dysfunction and subsequent adverse outcomes in AMI patients [45–48]. Such patients often have a higher prevalence of other chronic conditions, such as diabetes, hypertension, and renal dysfunction, which can adversely affect the management and outcomes of AMI [49]. Additionally, cancer-related factors such as tumor burden, metastasis, and treatment-related immunosuppression may make AMI patients more susceptible to adverse events and complications [50,51]. Furthermore, patients with cancer might have delayed or suboptimal access to cardiac care, as the focus of their healthcare may primarily revolve around cancer treatment. This delay in seeking medical attention or receiving appropriate cardiovascular interventions could also contribute to worse outcomes.

Our analysis demonstrated a higher risk of major bleeding in patients with AMI with previous or concurrent cancer. Cancer can lead to coagulation system abnormalities, such as thrombocytopenia, impaired platelet function, or alterations in clotting factors [44–46]. Additionally, cancer treatments, particularly anticoagulant therapies and antiplatelet agents, may further contribute to the increased bleeding risk [52]. The presence of cancer-related comorbidities, such as liver dysfunction or gastrointestinal involvement, can also enhance the propensity for bleeding in this population [53,54]. We also found an increased risk of reinfarction, which may be the result of cancer-related systemic inflammation, accelerated atherosclerosis, and impaired vascular healing, contributing to the destabilization of coronary plaques and subsequent reinfarction [43–46]. Moreover, the cardiotoxic effects of cancer treatments may further exacerbate the risk of recurrent ischemic events [47,48]. The meta-analysis identified an increased risk of stroke in this patient population. The mechanisms underlying this association could be related to hypercoagulability, endothelial dysfunction, and increased inflammatory markers, promoting a prothrombotic state and predisposing individuals to ischemic stroke [11,12,45,46].

It is important to acknowledge some limitations of this meta-analysis. First, the included studies were primarily observational (retrospective cohort design). While a retrospective study design can be a practical and suitable approach for addressing the research question dealt with in this meta-analysis, it has certain limitations. Retrospective studies help investigate long-term outcomes, large populations, and rare events like AMI in cancer patients, as they enable broad data collection from medical records or registries. However, they also pose challenges, such as potential biases (e.g., selection bias, recall bias), incomplete data, and difficulty controlling for confounders. In this analysis, the included studies employed appropriate confounder adjustments (e.g., multivariable analysis, propensity score matching). However, there is still a risk of some unmeasured and unadjusted confounding factors and consequent bias. Second, heterogeneity was observed for many of the outcomes. This variability may be due to variations in patient characteristics, study settings, differential definitions of the outcomes among studies, and treatments offered. Although we employed a random-effects model to account for this heterogeneity, caution should be exercised when interpreting the pooled results. Most of the included studies did not provide specific information about the type and stage of cancer or the mode of cancer management, which may have limited our overall analysis and contributed to heterogeneity in our results. Therefore, further high-quality studies are needed to validate the findings of our meta-analysis. Additionally, pooling studies with differing follow-up periods beyond the first year can be a limitation. Time-to-event data should be consistent across studies to ensure accurate estimates. However, the included studies had variable follow-up durations, which technically prevented pooling. Despite this, we opted to pool these findings to provide a fair indication of the risk. Another limitation is related to the fact that we were unable to perform subgroup analysis based on the type of AMI (i.e., ST-segment elevated MI (STEMI) and non-STEMI) because most studies predominantly reported data on patients with STEMI (N = 10) and some studies did not report the type of acute myocardial infarction at all (N = 7). Also, potential limitation of our study is the exclusion of studies focusing on specific subgroups of AMI, such as those with cardiogenic shock or cardiac arrest. While our objective was to synthesize data on the broader AMI population, we recognize that outcomes in these high-risk subgroups may differ significantly. This exclusion may limit the generalizability of our findings.

## Conclusion

Our study suggests that the presence of previous or concurrent cancer diagnoses in patients with AMI is associated with increased mortality and complications such as major bleeding, reinfarction, and stroke. Our findings highlight the importance of a multidisciplinary approach between oncologists, cardiologists, and other healthcare professionals to ensure comprehensive care that addresses both cancer-related concerns and cardiovascular health. Early cardioprotective interventions that are tailored to the specific needs of cancer patients can help mitigate the risk of MI and improve overall cardiovascular outcomes. Such interventions may include closely monitoring cardiac function, lifestyle modifications, and targeted pharmacological interventions. Further research is needed to investigate the specific mechanisms underlying these associations, as well as the impact of different cancer types, stages, and treatments on patient outcomes.

## Supporting information

S1 Fig Pooled risk of mortality at more than 1 year follow-up. (JPG)

S2 Fig Funnel plot for in-hospital mortality. (JPG)

S3 Fig Funnel plot for 30-day mortality. (JPG)

S4 Fig Funnel plot for mortality at 1 year of follow up. (JPG)

S5 Fig Pooled risk of in-hospital mortality among subgroup of patients undergoing PCI. (JPG)

S6 Fig Pooled risk of in-hospital mortality among subgroup of patients with prior cancer. (JPG)

S7 Fig Pooled risk of in-hospital mortality among subgroup of patients with concomitant cancer. (JPG)

S8 Fig Pooled risk of 30-day mortality among subgroup of patients undergoing PCI. (JPG)

S9 Fig Pooled risk of 30-day mortality among subgroup of patients with prior cancer. (JPG)

S10 Fig Pooled risk of 30-day mortality among subgroup of patients with concomitant cancer. (JPG)

S11 Fig Pooled risk of mortality at 1 year of follow-up among subgroup of patients undergoing PCI. (JPG)

S12 Fig Pooled risk of mortality at 1 year of follow-up among subgroup of patients with prior cancer. (JPG)

S13 Fig Pooled risk of mortality at 1 year of follow-up among subgroup of patients with concomitant cancer. (JPG)

S14 Fig Funnel plot for risk of major bleeding. (JPG)

S15 Fig Funnel plot for risk of reinfarction. (JPG)

S16 Fig Funnel plot for risk of stroke. (JPG)

S17 Fig Pooled risk of major bleeding among subgroup of patients undergoing PCI. (JPG)

S18 Fig Pooled risk of major bleeding among subgroup of patients with prior cancer. (JPG)

S19 Fig Pooled risk of major bleeding among subgroup of patients with concomitant cancer. (JPG)

S20 Fig Pooled risk of reinfarction among subgroup of patients undergoing PCI. (JPG)

S21 Fig Pooled risk of reinfarction among subgroup of patients with prior cancer. (JPG)

S22 Fig Pooled risk of reinfarction among subgroup of patients with concomitant cancer. (JPG)

S23 Fig Pooled risk of stroke among subgroup of patients undergoing PCI. (JPG)

S24 Fig Pooled risk of stroke among subgroup of patients with prior cancer. (JPG)

S25 Fig Pooled risk of stroke among subgroup of patients with concomitant cancer. (JPG)

S1 Table Search strategy in PubMed. (DOCX)

S2 Table Search strategy in EMBASE. (DOCX)

S3 Table Search strategy in Scopus. (DOCX)

S4 Table Author’s judgements about study quality using the Newcastle Ottawa Risk of Bias Assessment tool. (DOCX)

S5 Table Author’s judgements about study quality using the Newcastle Ottawa Risk of Bias Assessment tool. (DOCX)

S6 Table Quality of the pooled evidence using the GRADE assessment. (DOCX)

S1 File PRISMA checklist. (DOCX)

S2 File Excluded studies after full text review. (DOC)

## References

[1] Salari N, Morddarvanjoghi F, Abdolmaleki A, Rasoulpoor S, Khaleghi AA, Hezarkhani LA, et al. The global prevalence of myocardial infarction: a systematic review and meta-analysis. BMC Cardiovasc Disord. 2023;23: 206. doi: 10.1186/s12872-023-03231-w 37087452 PMC10122825

[2] Mattiuzzi C, Lippi G. Current Cancer Epidemiology. J Epidemiol Glob Health. 2019;9: 217–222. doi: 10.2991/jegh.k.191008.001 31854162 PMC7310786

[3] Sung H, Ferlay J, Siegel RL, Laversanne M, Soerjomataram I, Jemal A, et al. Global Cancer Statistics 2020: GLOBOCAN Estimates of Incidence and Mortality Worldwide for 36 Cancers in 185 Countries. CA Cancer J Clin. 2021;71: 209–249. doi: 10.3322/caac.21660 33538338

[4] Paterson DI, Wiebe N, Cheung WY, Mackey JR, Pituskin E, Reiman A, et al. Incident Cardiovascular Disease Among Adults With Cancer: A Population-Based Cohort Study. JACC CardioOncol. 2022;4: 85–94. doi: 10.1016/j.jaccao.2022.01.100 35492824 PMC9040097

[5] Yeh T-L, Hsu M-S, Hsu H-Y, Tsai M-C, Jhuang J-R, Chiang C-J, et al. Risk of cardiovascular diseases in cancer patients: A nationwide representative cohort study in Taiwan. BMC Cancer. 2022;22: 1198. doi: 10.1186/s12885-022-10314-y 36411401 PMC9677651

[6] Wang Y, Wang Y, Han X, Sun J, Li C, Adhikari BK, et al. Cardio-Oncology: A Myriad of Relationships Between Cardiovascular Disease and Cancer. Front Cardiovasc Med. 2022;9: 727487. doi: 10.3389/fcvm.2022.727487 35369296 PMC8968416

[7] Yang H, Bhoo-Pathy N, Brand JS, Hedayati E, Grassmann F, Zeng E, et al. Risk of heart disease following treatment for breast cancer—results from a population-based cohort study. Elife. 2022;11: e71562. doi: 10.7554/eLife.71562 35293856 PMC8940173

[8] Meijers WC, de Boer RA. Common risk factors for heart failure and cancer. Cardiovasc Res. 2019;115: 844–853. doi: 10.1093/cvr/cvz035 30715247 PMC6452432

[9] Johnson CB, Davis MK, Law A, Sulpher J. Shared Risk Factors for Cardiovascular Disease and Cancer: Implications for Preventive Health and Clinical Care in Oncology Patients. Can J Cardiol. 2016;32: 900–907. doi: 10.1016/j.cjca.2016.04.008 27343745

[10] Singh N, Baby D, Rajguru JP, Patil PB, Thakkannavar SS, Pujari VB. Inflammation and cancer. Ann Afr Med. 2019;18: 121–126. doi: 10.4103/aam.aam_56_18 31417011 PMC6704802

[11] Leiva O, AbdelHameid D, Connors JM, Cannon CP, Bhatt DL. Common Pathophysiology in Cancer, Atrial Fibrillation, Atherosclerosis, and Thrombosis: JACC: CardioOncology State-of-the-Art Review. JACC CardioOncol. 2021;3: 619–634. doi: 10.1016/j.jaccao.2021.08.011 34988471 PMC8702799

[12] Hamza MS, Mousa SA. Cancer-Associated Thrombosis: Risk Factors, Molecular Mechanisms, Future Management. Clin Appl Thromb Hemost. 2020;26: 1076029620954282. doi: 10.1177/1076029620954282 32877229 PMC7476343

[13] de Boer RA, Hulot J-S, Tocchetti CG, Aboumsallem JP, Ameri P, Anker SD, et al. Common mechanistic pathways in cancer and heart failure. A scientific roadmap on behalf of the Translational Research Committee of the Heart Failure Association (HFA) of the European Society of Cardiology (ESC). Eur J Heart Fail. 2020;22: 2272–2289. doi: 10.1002/ejhf.2029 33094495 PMC7894564

[14] Dongchen X, Tongyi L, Xueping M, Jingjing S, Quanhong L. Risk of mortality and other adverse outcomes from myocardial infarction in cancer survivors: a meta-analysis. Int J Clin Oncol. 2023;28: 41–51. doi: 10.1007/s10147-022-02276-9 36443616

[15] Machanahalli Balakrishna A, Ismayl M, Srinivasamurthy R, Gowda RM, Aboeata A. Early Outcomes of Percutaneous Coronary Intervention in Patients with Cancer: A Systematic Review and Meta-analysis. Curr Probl Cardiol. 2022;47: 101305. doi: 10.1016/j.cpcardiol.2022.101305 35798277

[16] PRISMA. Transparent reporting of systematic reviews and meta-analyses. Available: http://www.prisma-statement.org/

[17] Wells G, Shea B, O’Connell D, Robertson J, Peterson J, Losos M, et al. The Newcastle-Ottawa Scale (NOS) for Assessing the Quality of Nonrandomized Studies in Meta- Analysis.

[18] Cochrane Handbook for Systematic Reviews of Interventions. [cited 12 Feb 2023]. Available: https://training.cochrane.org/handbook

[19] Egger M, Davey Smith G, Schneider M, Minder C. Bias in meta-analysis detected by a simple, graphical test. BMJ. 1997;315: 629–634. doi: 10.1136/bmj.315.7109.629 9310563 PMC2127453

[20] McMaster University and Evidence Prime. GRADEpro GDT: GRADEpro Guideline Development Tool [Software]. [cited 8 Aug 2024]. Available: https://www.gradepro.org/

[21] Dafaalla M, Abdel-Qadir H, Gale CP, Sun L, López-Fernández T, Miller RJH, et al. Outcomes of ST elevation myocardial infarction in patients with cancer; a nationwide study. Eur Heart J Qual Care Clin Outcomes. 2023; qcad012. doi: 10.1093/ehjqcco/qcad012 36921979

[22] Hayashi H, Kataoka Y, Murai K, Sawada K, Iwai T, Matama H, et al. Cardiovascular and bleeding risks of inactive cancer in patients with acute myocardial infarction who received primary percutaneous coronary intervention using drug-eluting stent and dual/triple antithrombotic therapy. Cardiovasc Diagn Ther. 2022;12: 803–814. doi: 10.21037/cdt-22-306 36605075 PMC9808111

[23] Koo CY, Zheng H, Tan LL, Foo L-L, Hausenloy DJ, Chng W-J, et al. Prior Cancer Is Associated with Lower Atherosclerotic Cardiovascular Disease Risk at First Acute Myocardial Infarction. Biomedicines. 2022;10: 2681. doi: 10.3390/biomedicines10112681 36359201 PMC9687197

[24] Nozaka M, Yokoyama H, Kitayama K, Nagawa D, Hamadate M, Miura N, et al. Clinical Outcomes of Acute Myocardial Infarction Patients With a History of Malignant Tumor. In Vivo. 2020;34: 3589–3595. doi: 10.21873/invivo.12203 33144472 PMC7811638

[25] Takeuchi T, Kosugi S, Ueda Y, Ikeoka K, Yamane H, Takayasu K, et al. Impact of a Cancer History on Cardiovascular Events Among Patients With Myocardial Infarction Who Received Revascularization. Circ J. 2023. doi: 10.1253/circj.CJ-22-0838 37045768

[26] Ye J, Zhang L, Lyu J, Wang Y, Yuan S, Qin Z, et al. Malignant cancer may increase the risk of all-cause in-hospital mortality in patients with acute myocardial infarction: a multicenter retrospective study of two large public databases. Cardiooncology. 2023;9: 6. doi: 10.1186/s40959-023-00156-3 36670511 PMC9862556

[27] Velders MA, Boden H, Hofma SH, Osanto S, van der Hoeven BL, Heestermans AACM, et al. Outcome after ST elevation myocardial infarction in patients with cancer treated with primary percutaneous coronary intervention. Am J Cardiol. 2013;112: 1867–1872. doi: 10.1016/j.amjcard.2013.08.019 24063839

[28] Wang F, Gulati R, Lennon RJ, Lewis BR, Park J, Sandhu GS, et al. Cancer History Portends Worse Acute and Long-term Noncardiac (but Not Cardiac) Mortality After Primary Percutaneous Coronary Intervention for Acute ST-Segment Elevation Myocardial Infarction. Mayo Clin Proc. 2016;91: 1680–1692. doi: 10.1016/j.mayocp.2016.06.029 27916154

[29] Landes U, Kornowski R, Bental T, Assali A, Vaknin-Assa H, Lev E, et al. Long-term outcomes after percutaneous coronary interventions in cancer survivors. Coron Artery Dis. 2017;28: 5–10. doi: 10.1097/MCA.0000000000000429 27622995

[30] Iannaccone M D’Ascenzo F, Vadalà P, Wilton SB, Noussan P, Colombo F, et al. Prevalence and outcome of patients with cancer and acute coronary syndrome undergoing percutaneous coronary intervention: a BleeMACS substudy. Eur Heart J Acute Cardiovasc Care. 2018;7: 631–638. doi: 10.1177/2048872617706501 28593789

[31] Nakatsuma K, Shiomi H, Morimoto T, Watanabe H, Nakagawa Y, Furukawa Y, et al. Influence of a history of cancer on long-term cardiovascular outcomes after coronary stent implantation (an Observation from Coronary Revascularization Demonstrating Outcome Study-Kyoto Registry Cohort-2). Eur Heart J Qual Care Clin Outcomes. 2018;4: 200–207. doi: 10.1093/ehjqcco/qcy014 29897437

[32] Potts J, Mohamed MO, Lopez Mattei JC, Iliescu CA, Konopleva M, Rashid M, et al. Percutaneous coronary intervention and in-hospital outcomes in patients with leukemia: a nationwide analysis. Catheter Cardiovasc Interv. 2020;96: 53–63. doi: 10.1002/ccd.28432 31410970

[33] Guo W, Fan X, Lewis BR, Johnson MP, Rihal CS, Lerman A, et al. Cancer Patients Have a Higher Risk of Thrombotic and Ischemic Events After Percutaneous Coronary Intervention. JACC Cardiovasc Interv. 2021;14: 1094–1105. doi: 10.1016/j.jcin.2021.03.049 34016406 PMC8841226

[34] Peng X, Wang Z, Cao M, Zheng Y, Tian Y, Yu L, et al. A Concomitant Cancer Diagnosis Is Associated With Poor Cardiovascular Outcomes Among Acute Myocardial Infarction Patients. Front Cardiovasc Med. 2022;9: 758324. doi: 10.3389/fcvm.2022.758324 35252376 PMC8891500

[35] Tosaka K, Ishida M, Tsuji K, Kanehama N, Koeda Y, Niiyama M, et al. Prevalence, clinical characteristics, and impact of active cancer in patients with acute myocardial infarction: data from an all-comer registry. J Cardiol. 2021;78: 193–200. doi: 10.1016/j.jjcc.2021.04.004 34167885

[36] Zheng R, Kusunose K, Okushi Y, Okayama Y, Nakai M, Sumita Y, et al. Impact of cancer on short-term in-hospital mortality after primary acute myocardial infarction. Open Heart. 2021;8: e001860. doi: 10.1136/openhrt-2021-001860 34810277 PMC8609927

[37] Bharadwaj A, Potts J, Mohamed MO, Parwani P, Swamy P, Lopez-Mattei JC, et al. Acute myocardial infarction treatments and outcomes in 6.5 million patients with a current or historical diagnosis of cancer in the USA. Eur Heart J. 2020;41: 2183–2193. doi: 10.1093/eurheartj/ehz851 31800032

[38] Ederhy S, Cohen A, Boccara F, Puymirat E, Aissaoui N, Elbaz M, et al. In-hospital outcomes and 5-year mortality following an acute myocardial infarction in patients with a history of cancer: Results from the French registry on Acute ST-elevation or non-ST-elevation myocardial infarction (FAST-MI) 2005 cohort. Arch Cardiovasc Dis. 2019;112: 657–669. doi: 10.1016/j.acvd.2019.06.012 31761740

[39] Itzhaki Ben Zadok O, Hasdai D, Gottlieb S, Porter A, Beigel R, Shimony A, et al. Characteristics and outcomes of patients with cancer presenting with acute myocardial infarction. Coron Artery Dis. 2019;30: 332–338. doi: 10.1097/MCA.0000000000000733 30883428

[40] Gong IY, Yan AT, Ko DT, Earle CC, Cheung WY, Peacock S, et al. Temporal changes in treatments and outcomes after acute myocardial infarction among cancer survivors and patients without cancer, 1995 to 2013. Cancer. 2018;124: 1269–1278. doi: 10.1002/cncr.31174 29211307 PMC7614832

[41] Kurisu S, Iwasaki T, Ishibashi K, Mitsuba N, Dohi Y, Kihara Y. Comparison of treatment and outcome of acute myocardial infarction between cancer patients and non-cancer patients. Int J Cardiol. 2013;167: 2335–2337. doi: 10.1016/j.ijcard.2012.11.009 23201078

[42] Velders MA, Hagström E, James SK. Temporal Trends in the Prevalence of Cancer and Its Impact on Outcome in Patients With First Myocardial Infarction: A Nationwide Study. J Am Heart Assoc. 2020;9: e014383. doi: 10.1161/JAHA.119.014383 32067596 PMC7070202

[43] Noble S, Pasi J. Epidemiology and pathophysiology of cancer-associated thrombosis. Br J Cancer. 2010;102 Suppl 1: S2–9. doi: 10.1038/sj.bjc.6605599 20386546 PMC3315367

[44] Abdol Razak NB, Jones G, Bhandari M, Berndt MC, Metharom P. Cancer-Associated Thrombosis: An Overview of Mechanisms, Risk Factors, and Treatment. Cancers (Basel). 2018;10: 380. doi: 10.3390/cancers10100380 30314362 PMC6209883

[45] Grover SP, Hisada YM, Kasthuri RS, Reeves BN, Mackman N. Cancer Therapy-Associated Thrombosis. Arterioscler Thromb Vasc Biol. 2021;41: 1291–1305. doi: 10.1161/ATVBAHA.120.314378 33567864 PMC7990713

[46] Herrmann J. Vascular toxic effects of cancer therapies. Nat Rev Cardiol. 2020;17: 503–522. doi: 10.1038/s41569-020-0347-2 32218531 PMC8782612

[47] Chen ZI, Ai DI. Cardiotoxicity associated with targeted cancer therapies. Mol Clin Oncol. 2016;4: 675–681. doi: 10.3892/mco.2016.800 27123262 PMC4840776

[48] Ruddy KJ, Patel SR, Higgins AS, Armenian SH, Herrmann J. Cardiovascular Health during and after Cancer Therapy. Cancers (Basel). 2020;12: 3737. doi: 10.3390/cancers12123737 33322622 PMC7763346

[49] Fowler H, Belot A, Ellis L, Maringe C, Luque-Fernandez MA, Njagi EN, et al. Comorbidity prevalence among cancer patients: a population-based cohort study of four cancers. BMC Cancer. 2020;20: 2. doi: 10.1186/s12885-019-6472-9 31987032 PMC6986047

[50] Owusu C, Berger NA. Comprehensive geriatric assessment in the older cancer patient: coming of age in clinical cancer care. Clin Pract (Lond). 2014;11: 749–762. doi: 10.2217/cpr.14.72 25642321 PMC4308946

[51] Ness KK, Wogksch MD. Frailty and aging in cancer survivors. Transl Res. 2020;221: 65–82. doi: 10.1016/j.trsl.2020.03.013 32360946 PMC7321876

[52] Al-Samkari H, Connors JM. Managing the competing risks of thrombosis, bleeding, and anticoagulation in patients with malignancy. Blood Adv. 2019;3: 3770–3779. doi: 10.1182/bloodadvances.2019000369 31770442 PMC6880899

[53] Johnstone C, Rich SE. Bleeding in cancer patients and its treatment: a review. Ann Palliat Med. 2018;7: 265–273. doi: 10.21037/apm.2017.11.01 29307210

[54] Angelini DE, Radivoyevitch T, McCrae KR, Khorana AA. Bleeding incidence and risk factors among cancer patients treated with anticoagulation. Am J Hematol. 2019;94: 780–785. doi: 10.1002/ajh.25494 31006890

