# Supplementary figures and images for: A meta-analysis on the impact of concurrent or pre-existing cancer diagnosis on acute myocardial infarction outcomes
Source: PLoS One. 2025 Jan 31;20(1):e0318437. doi: 10.1371/journal.pone.0318437 (PMC11785289; doi:10.1371/journal.pone.0318437)

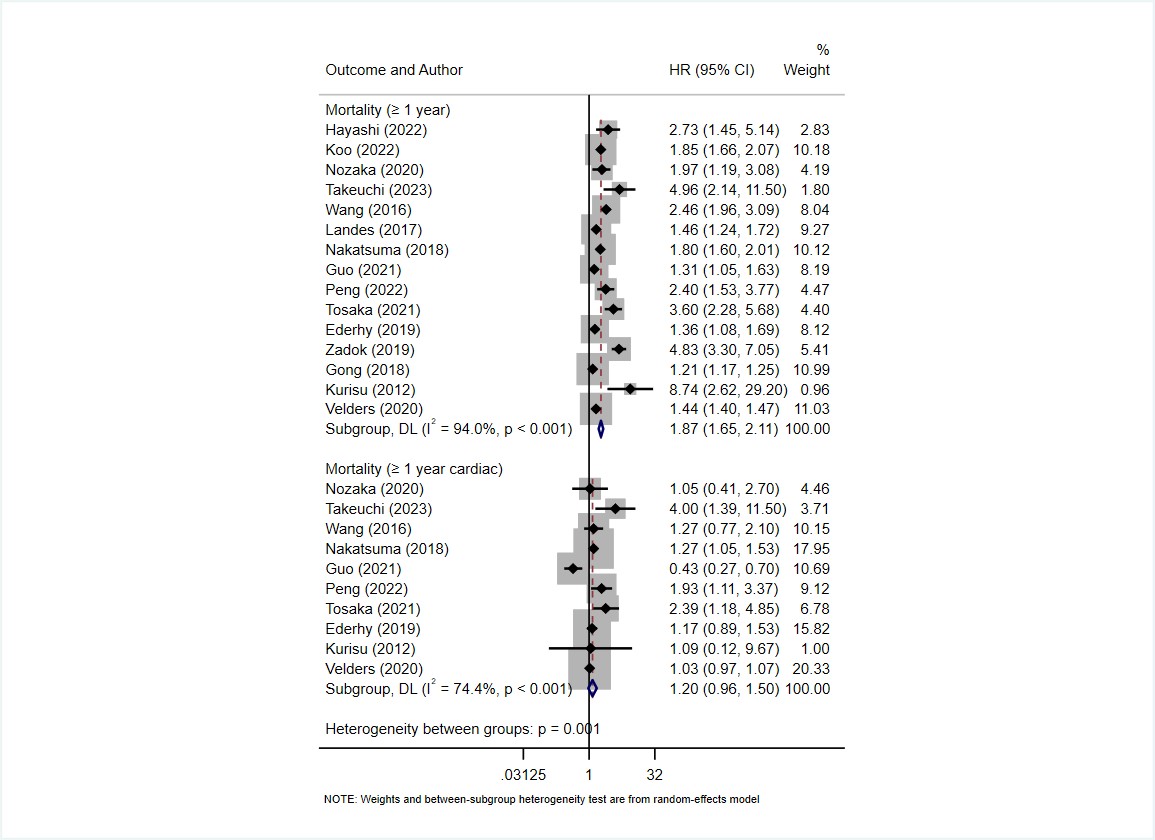

Supplement: S1 Fig — (JPG) [file pone.0318437.s001.jpg]

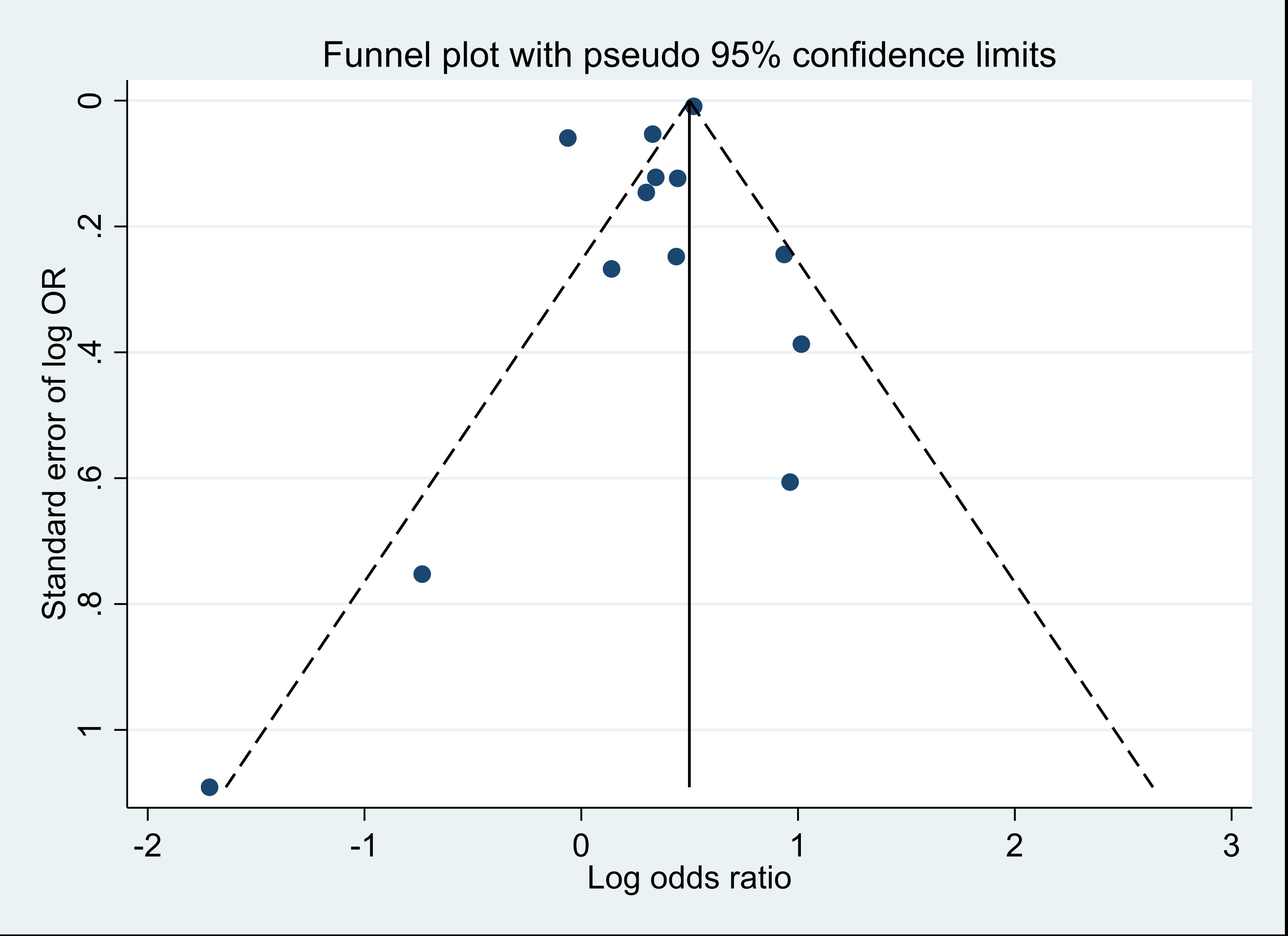

Supplement: S2 Fig — (JPG) [file pone.0318437.s002.jpg]

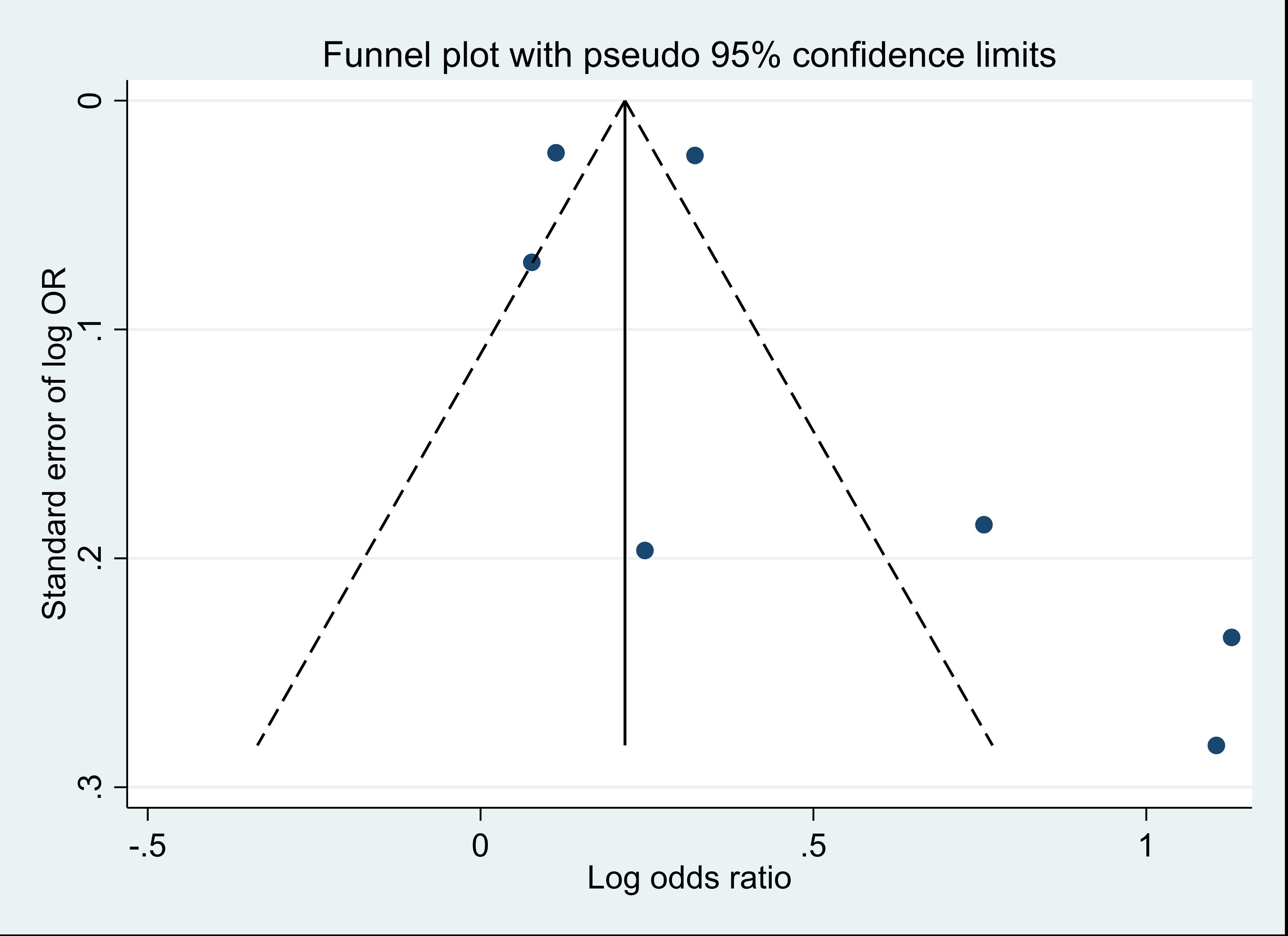

Supplement: S3 Fig — (JPG) [file pone.0318437.s003.jpg]

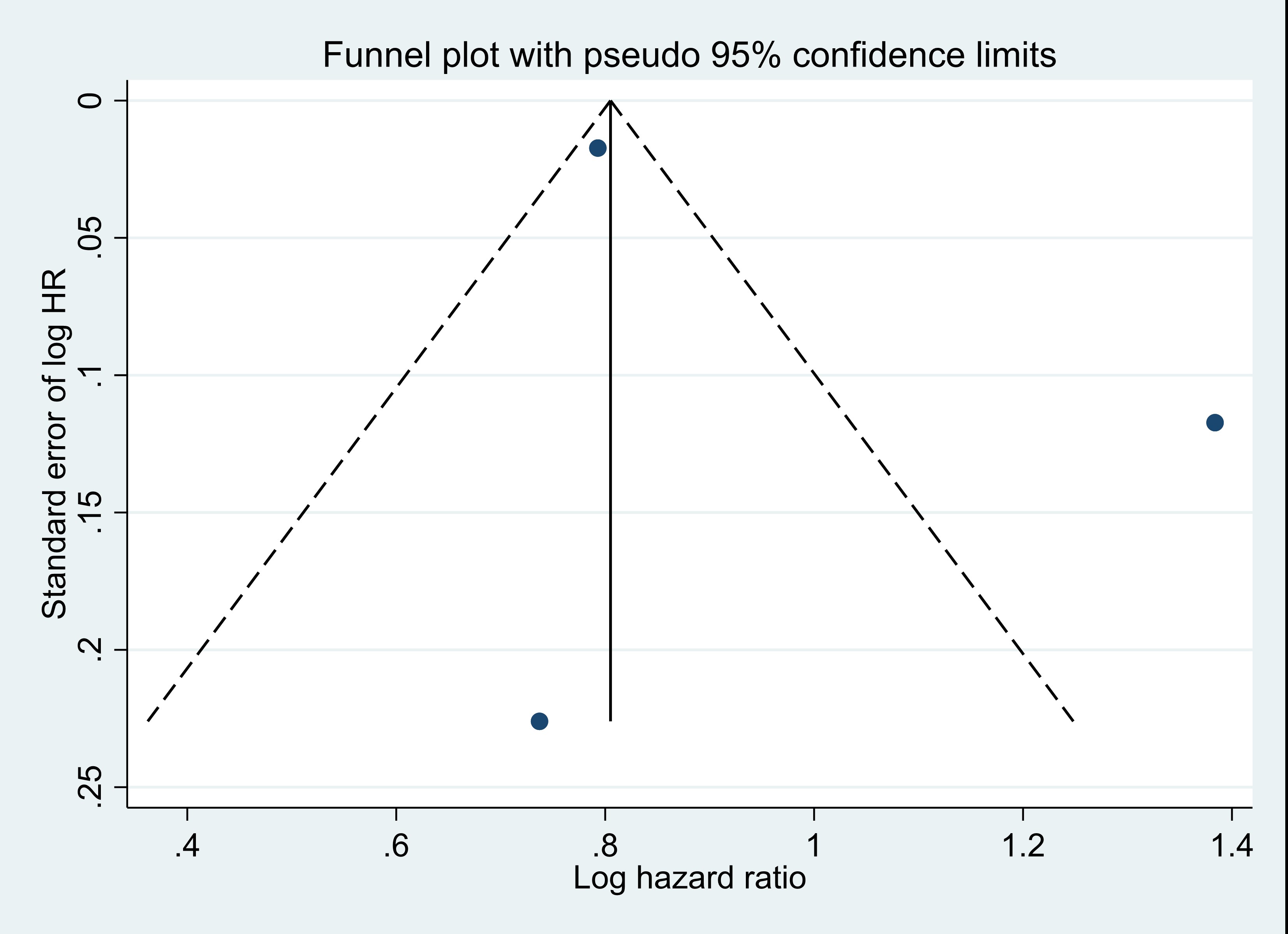

Supplement: S4 Fig — (JPG) [file pone.0318437.s004.jpg]

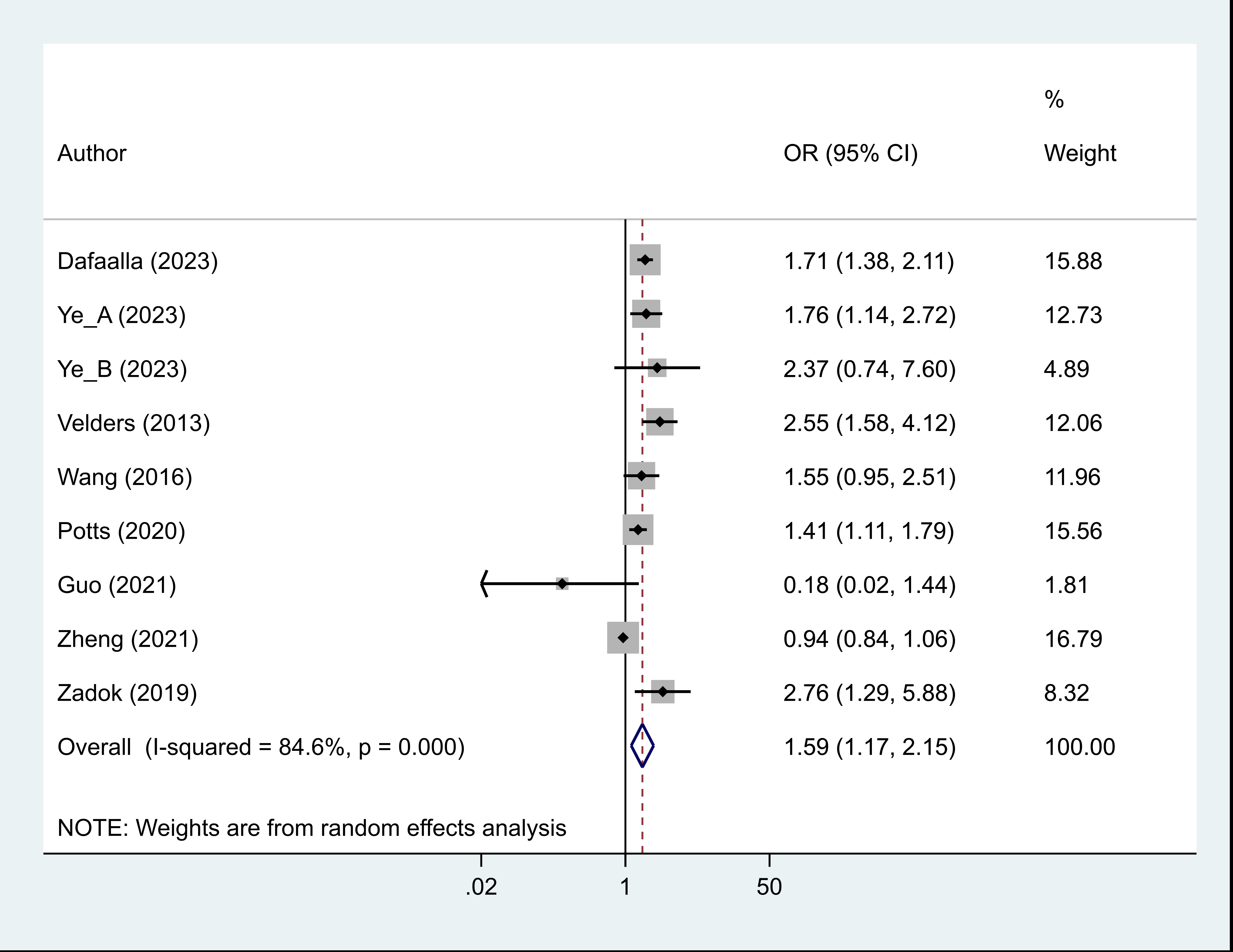

Supplement: S5 Fig — (JPG) [file pone.0318437.s005.jpg]

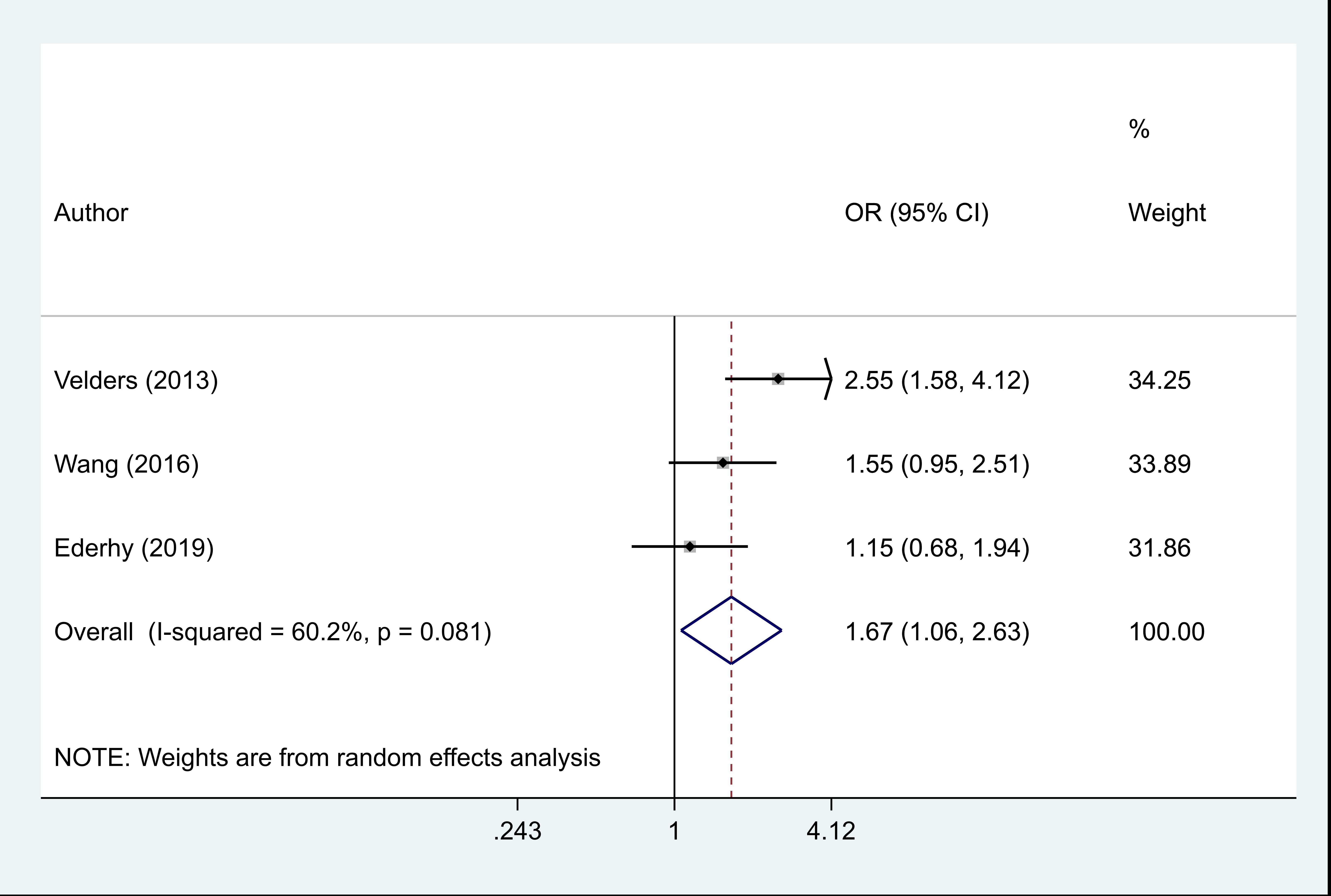

Supplement: S6 Fig — (JPG) [file pone.0318437.s006.jpg]

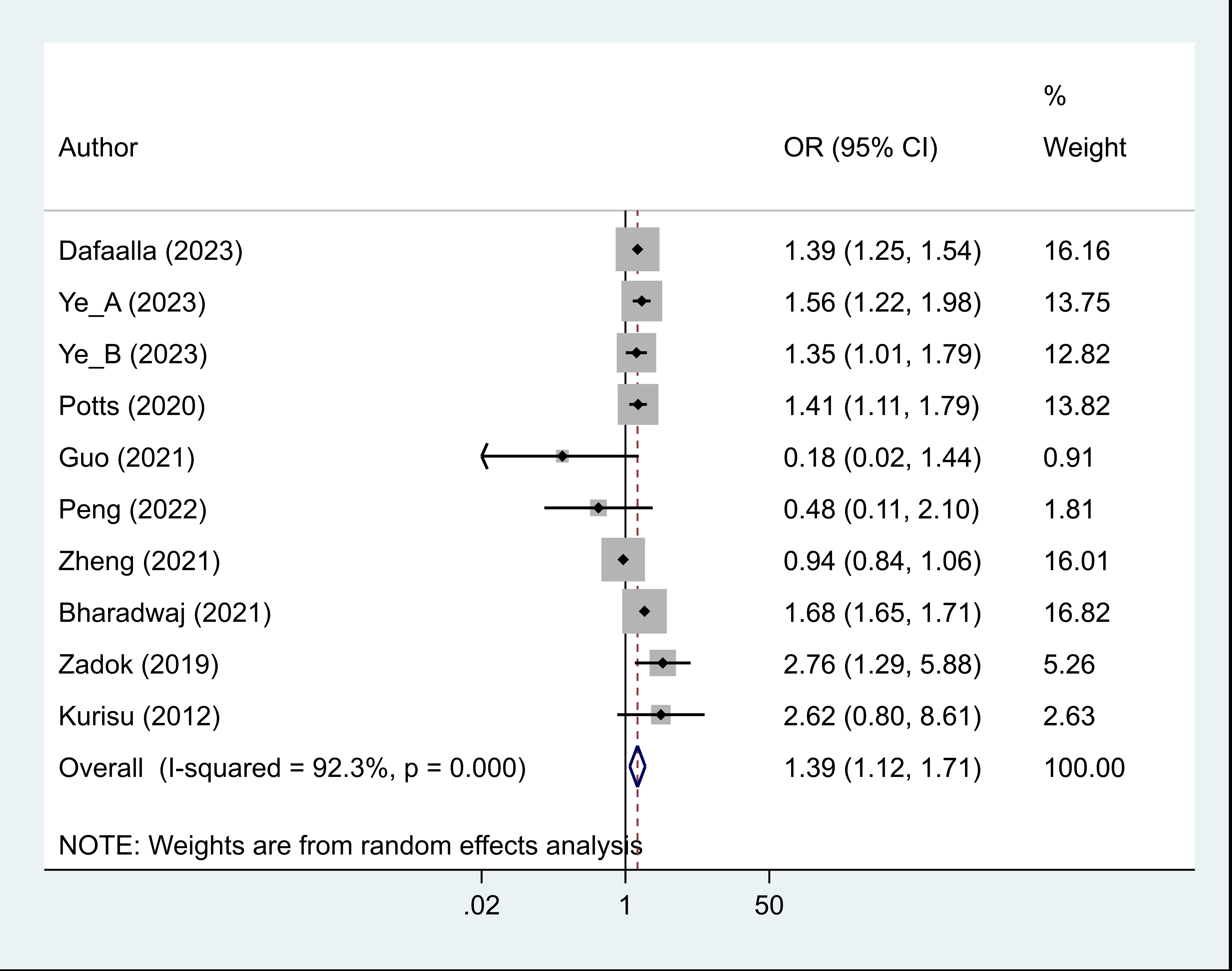

Supplement: S7 Fig — (JPG) [file pone.0318437.s007.jpg]

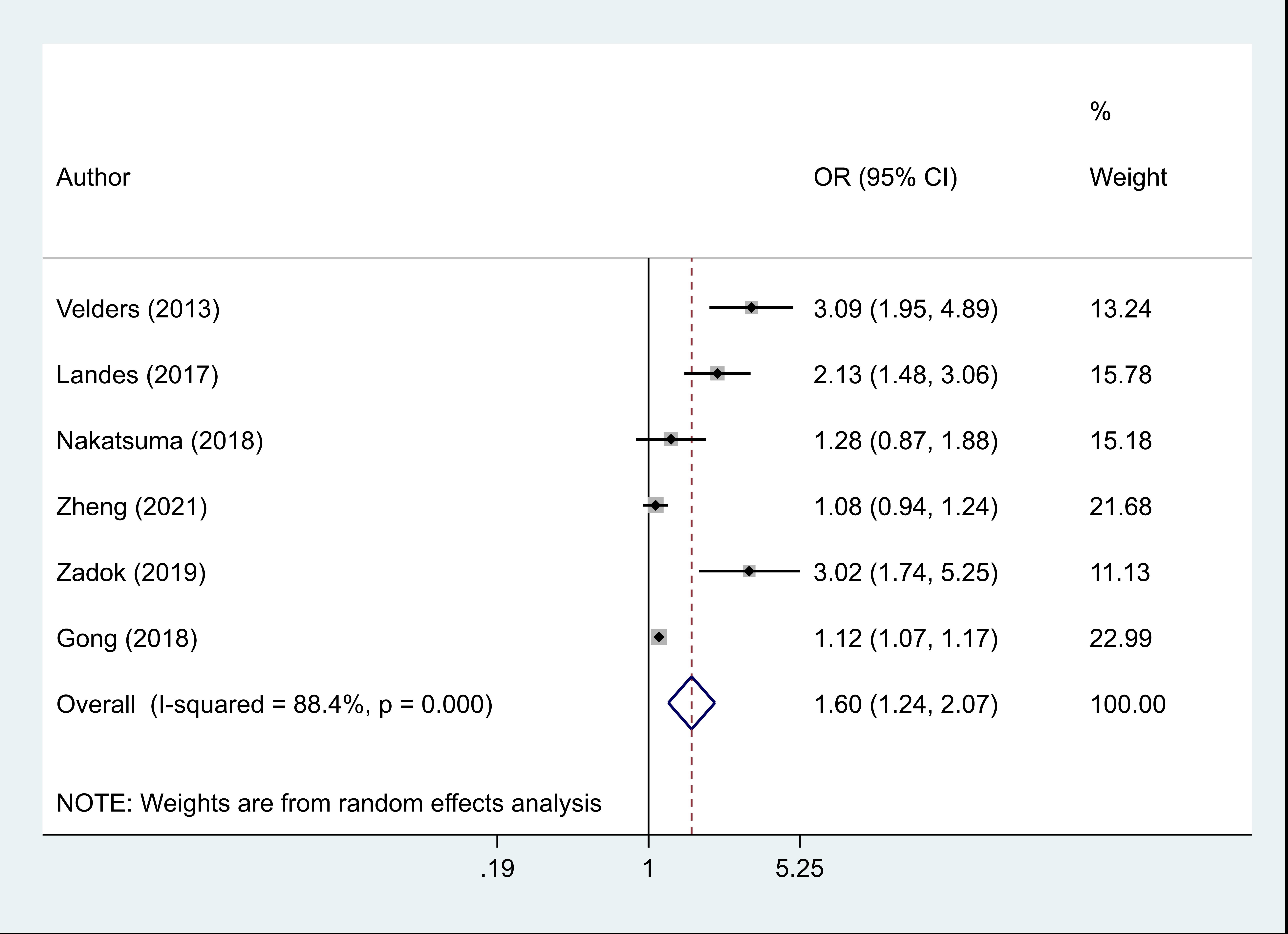

Supplement: S8 Fig — (JPG) [file pone.0318437.s008.jpg]

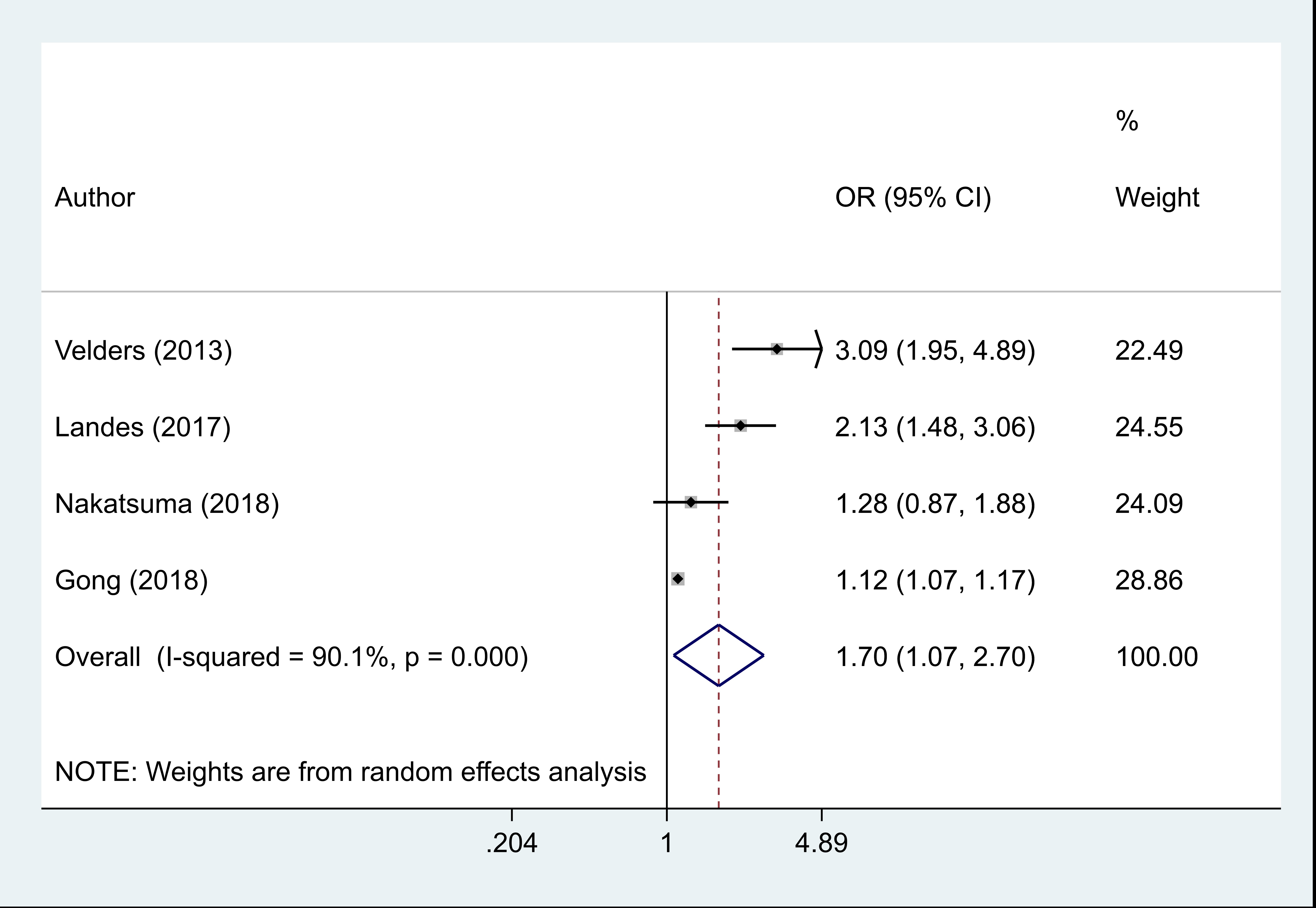

Supplement: S9 Fig — (JPG) [file pone.0318437.s009.jpg]

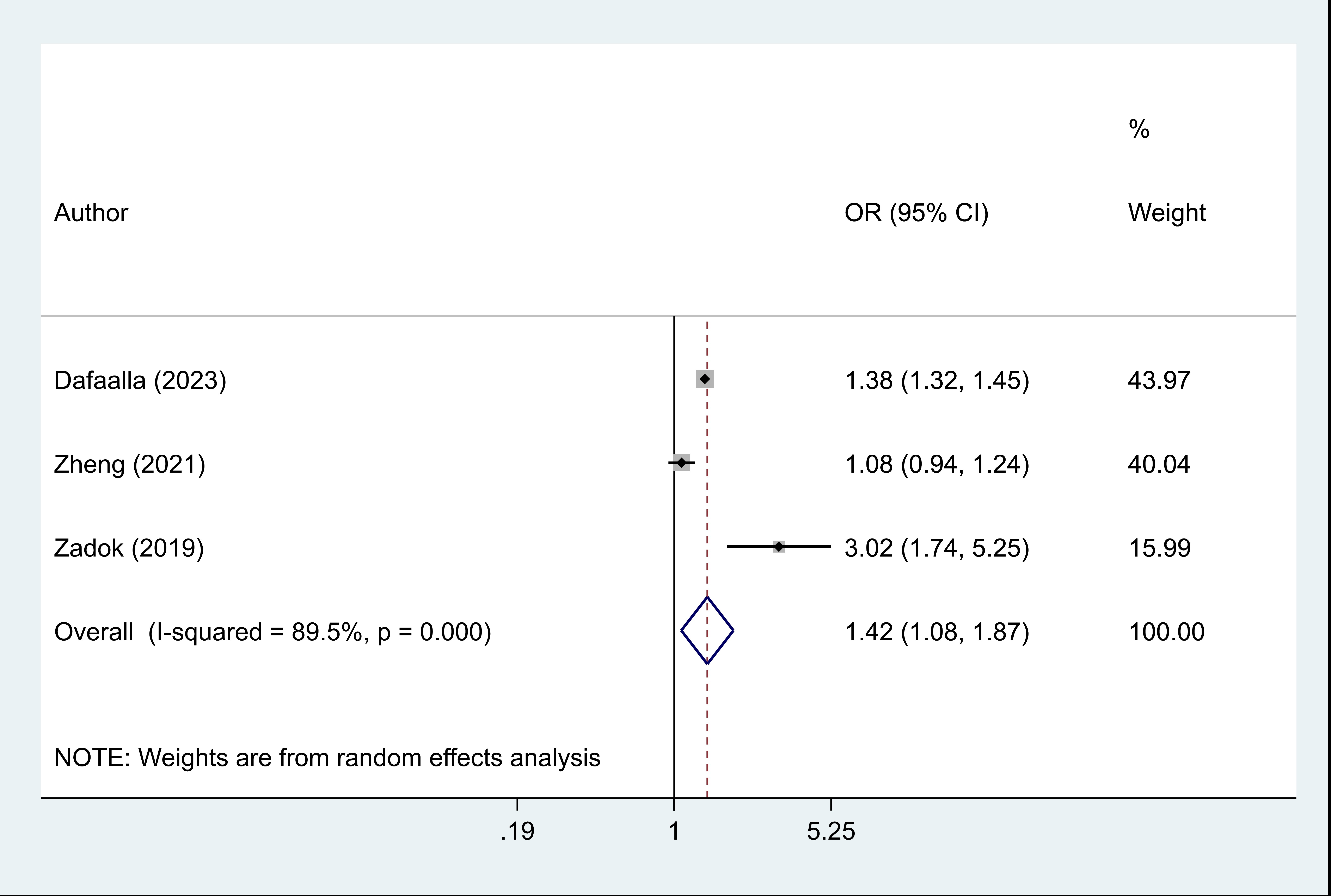

Supplement: S10 Fig — (JPG) [file pone.0318437.s010.jpg]

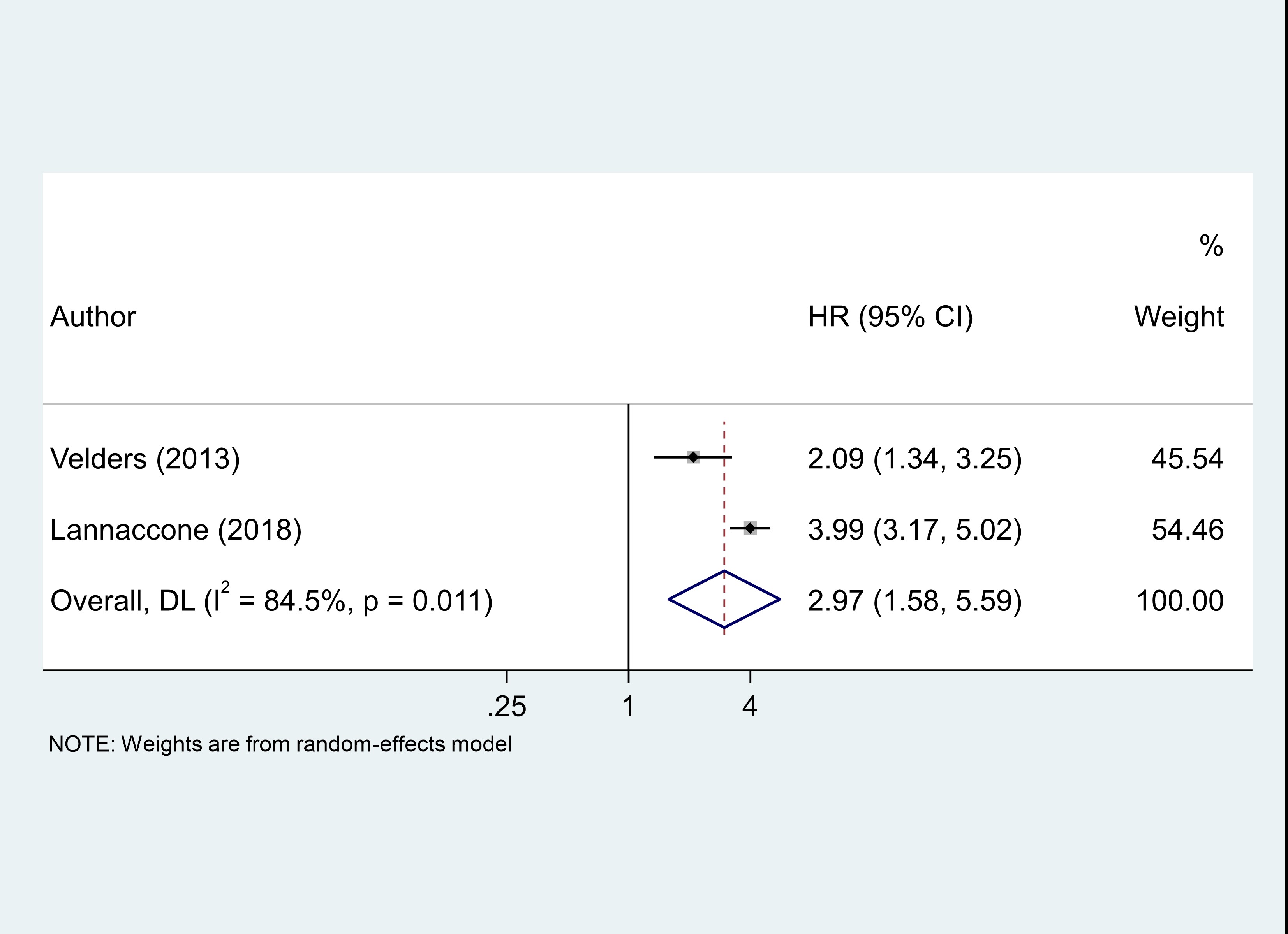

Supplement: S11 Fig — (JPG) [file pone.0318437.s011.jpg]

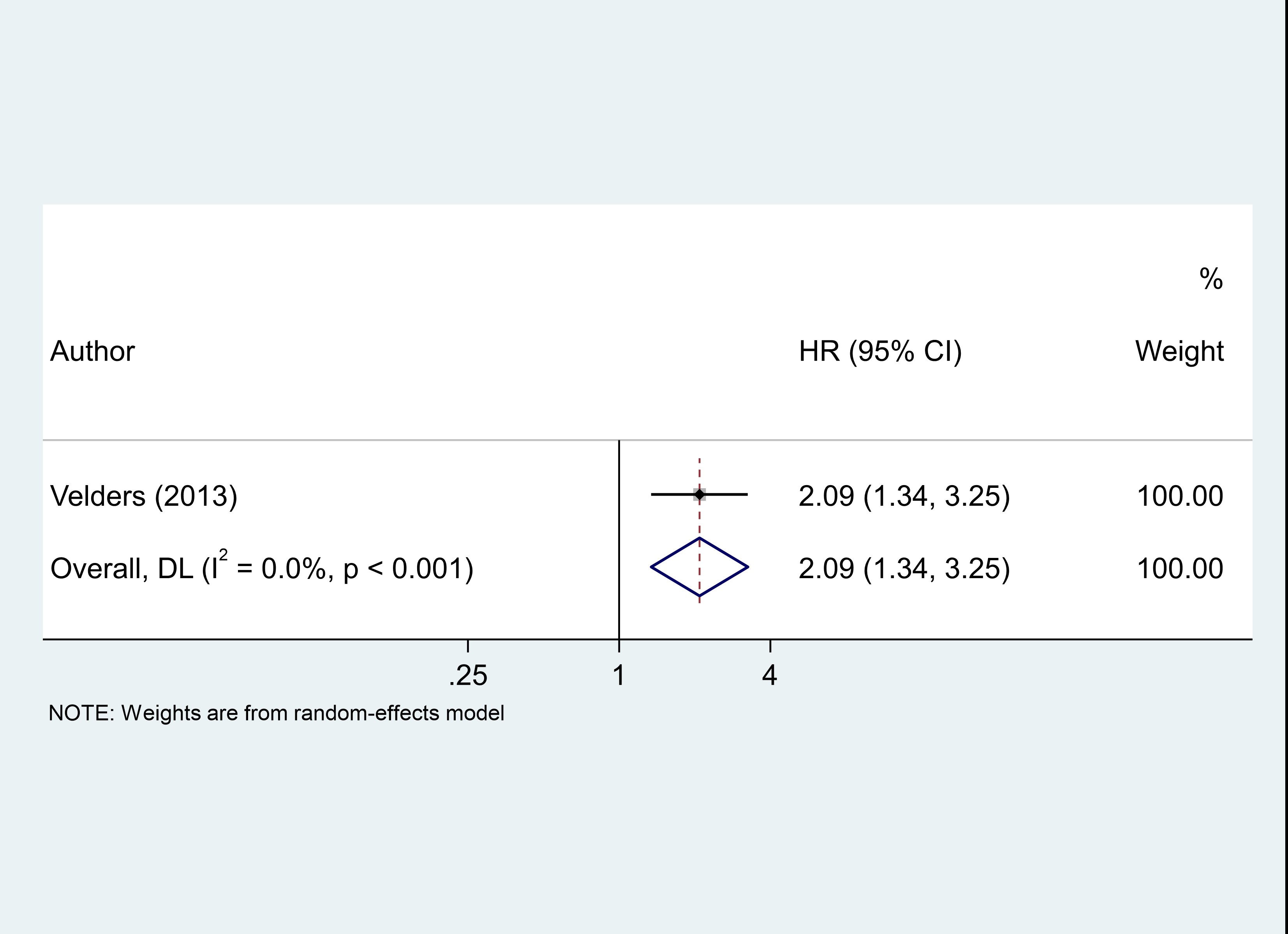

Supplement: S12 Fig — (JPG) [file pone.0318437.s012.jpg]

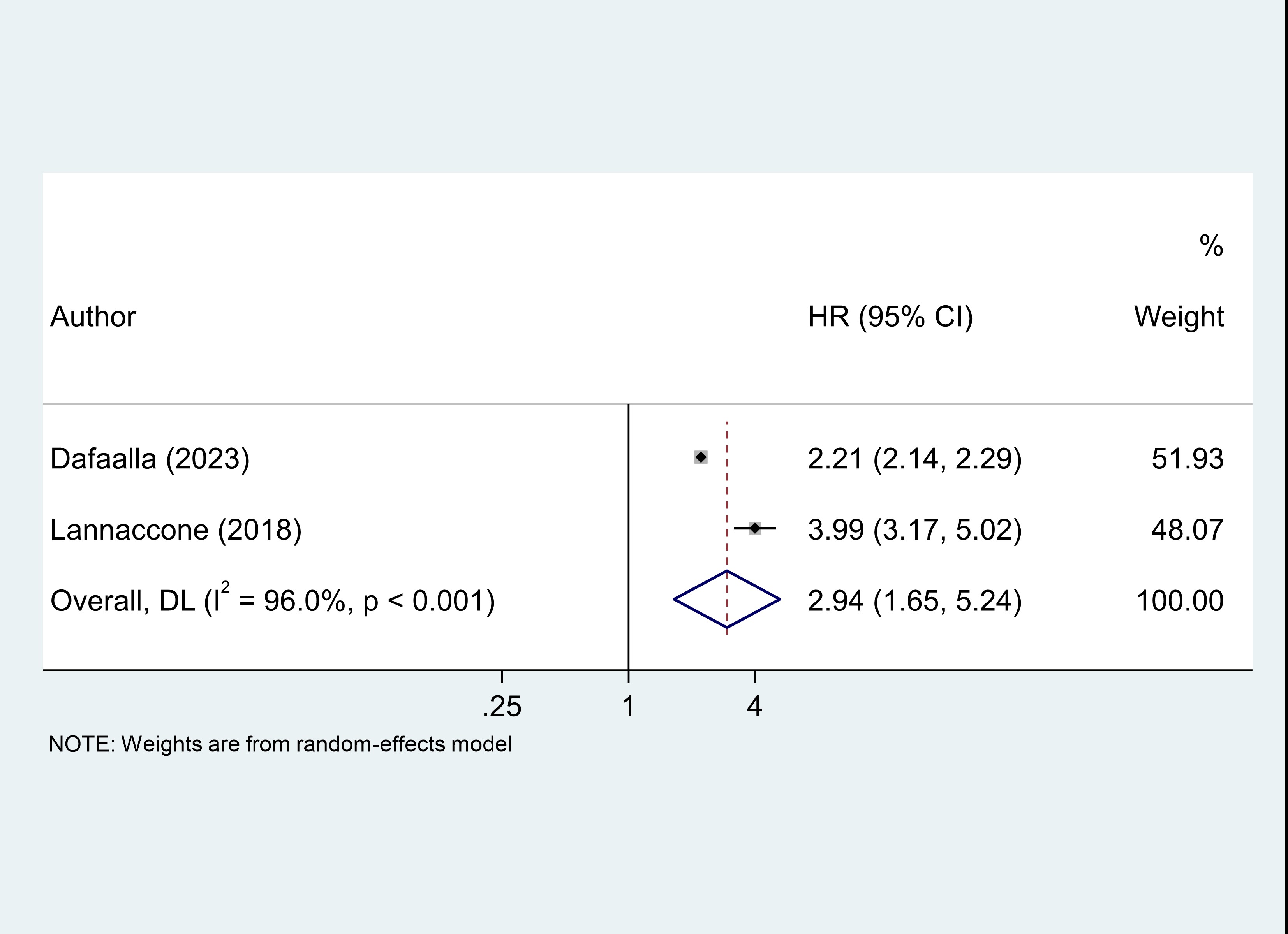

Supplement: S13 Fig — (JPG) [file pone.0318437.s013.jpg]

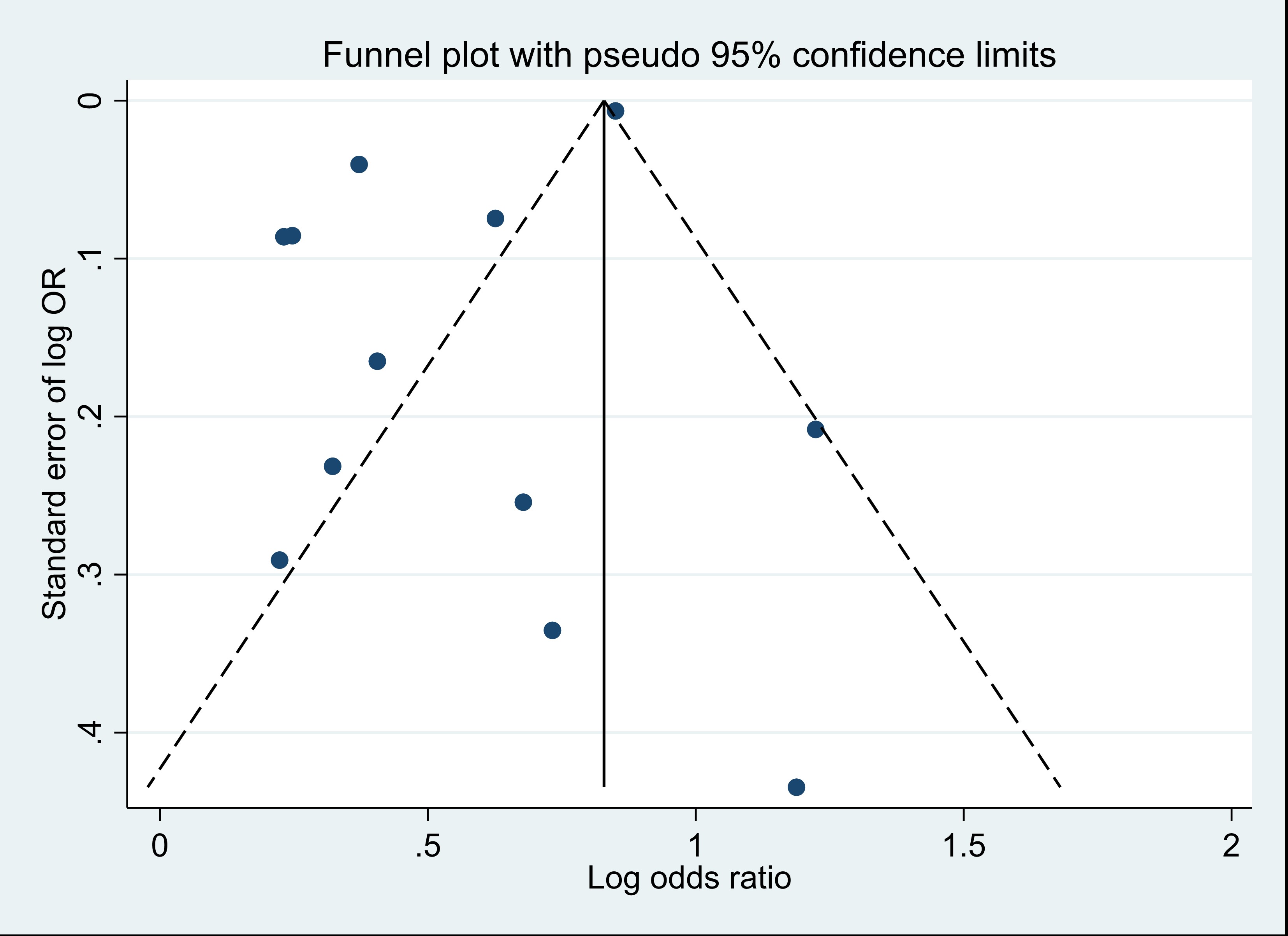

Supplement: S14 Fig — (JPG) [file pone.0318437.s014.jpg]

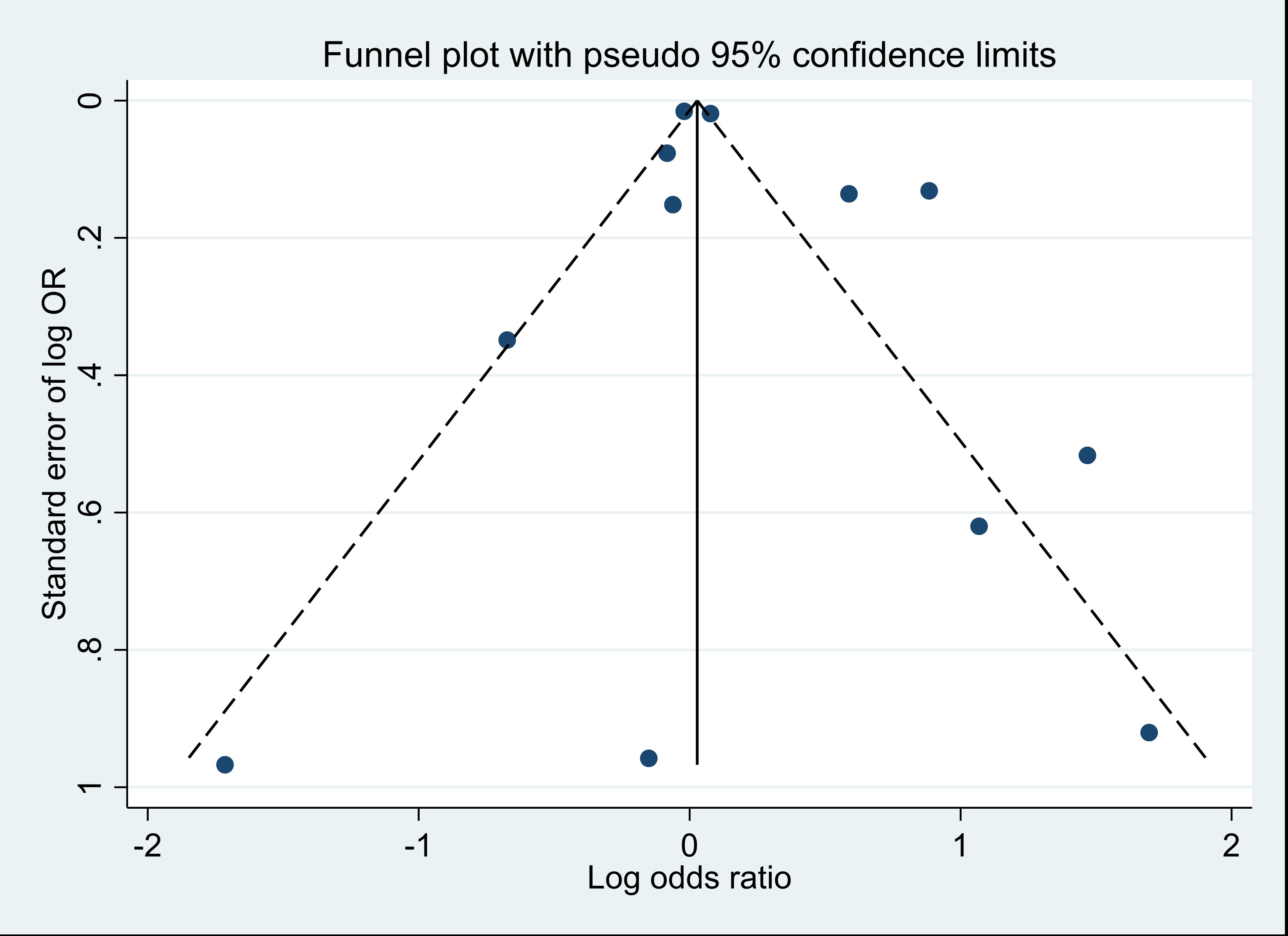

Supplement: S15 Fig — (JPG) [file pone.0318437.s015.jpg]

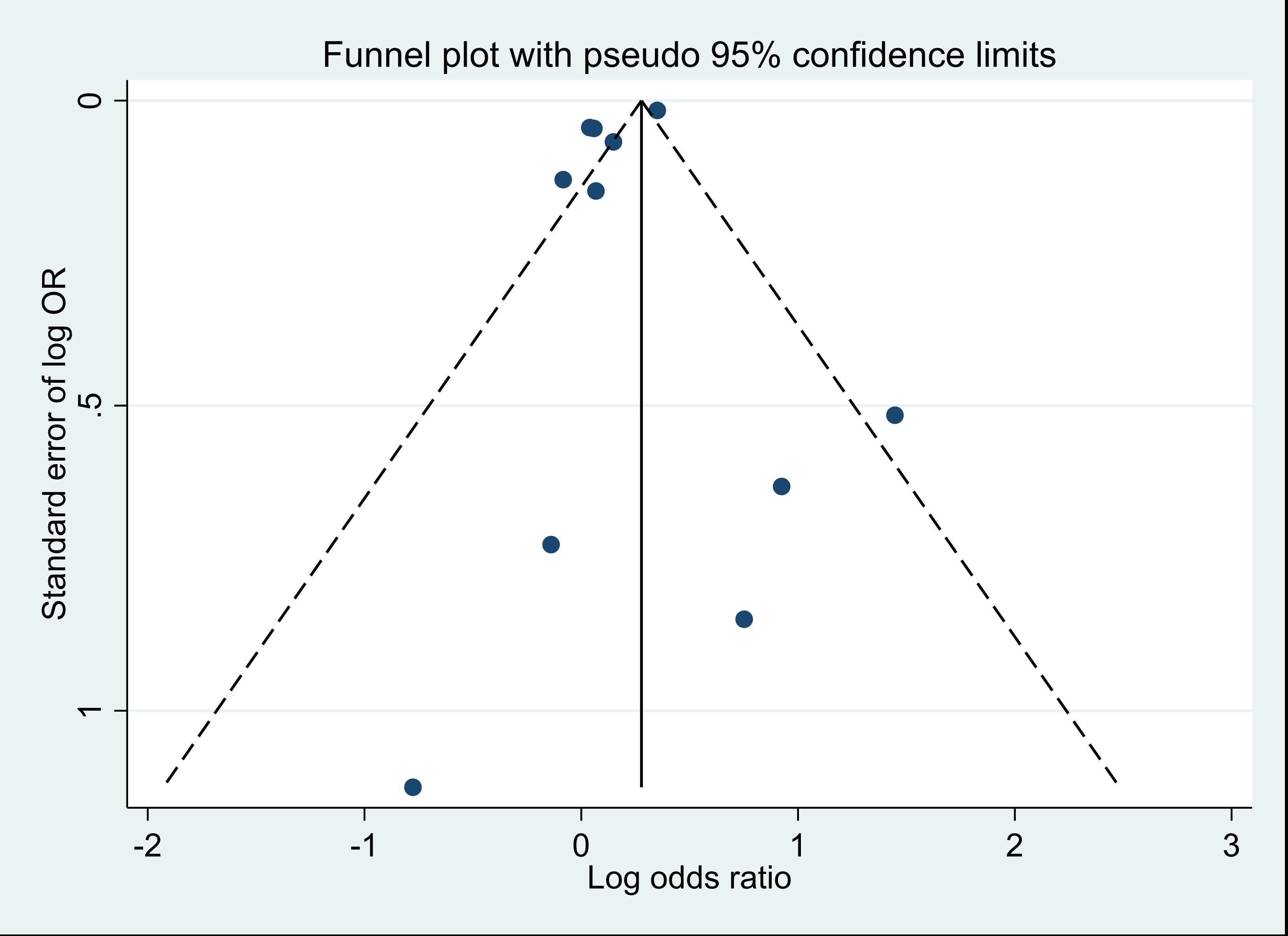

Supplement: S16 Fig — (JPG) [file pone.0318437.s016.jpg]

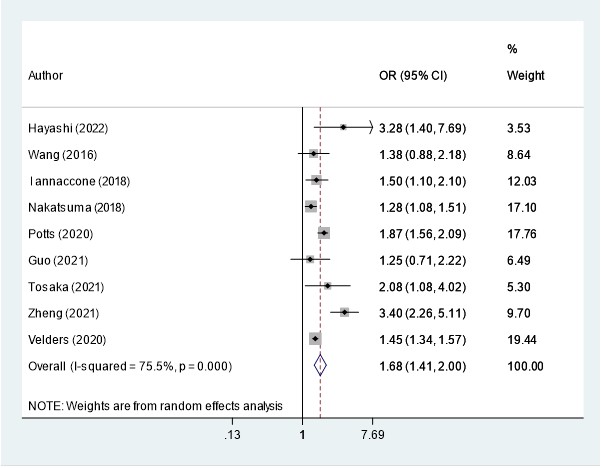

Supplement: S17 Fig — (JPG) [file pone.0318437.s017.jpg]

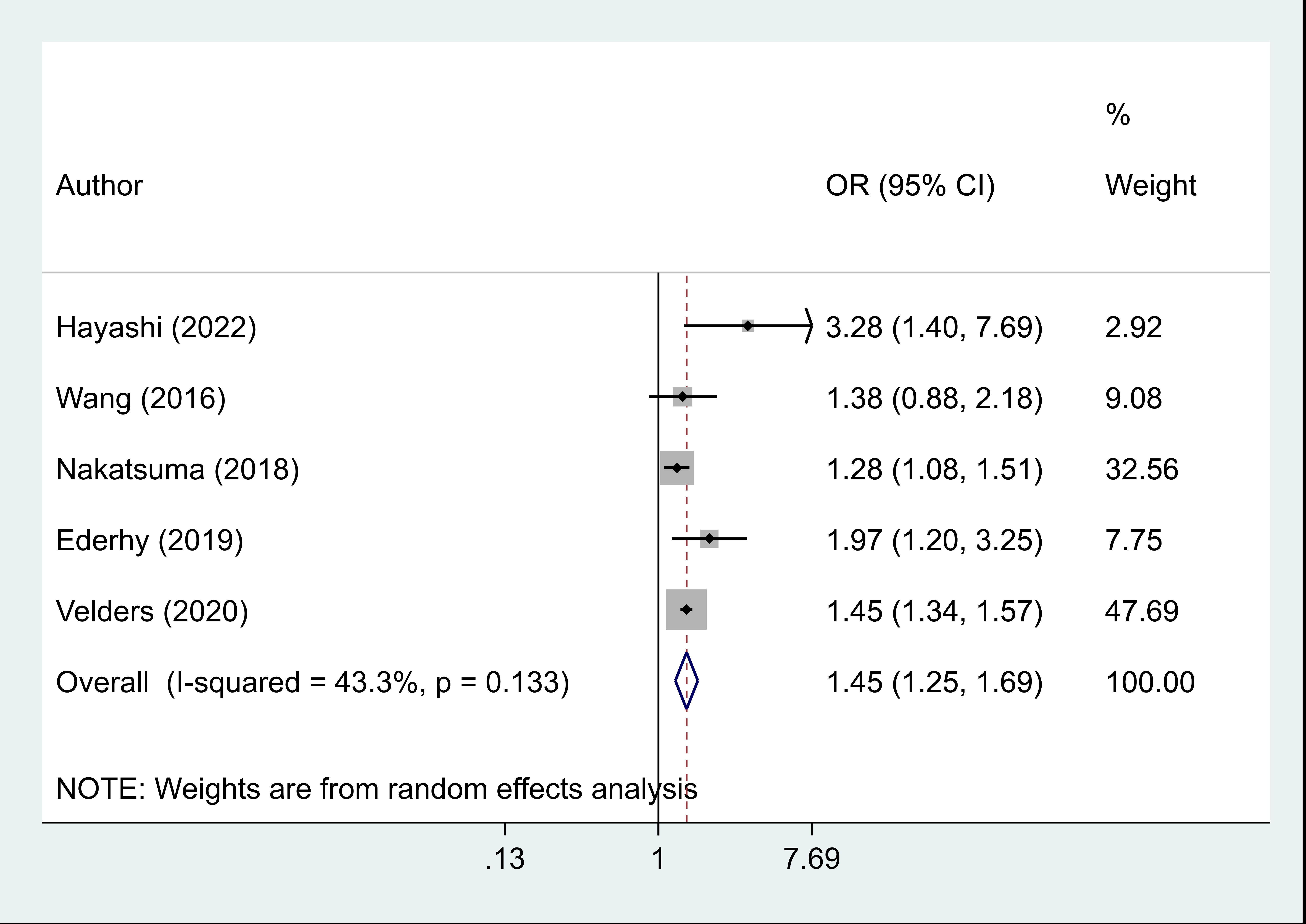

Supplement: S18 Fig — (JPG) [file pone.0318437.s018.jpg]

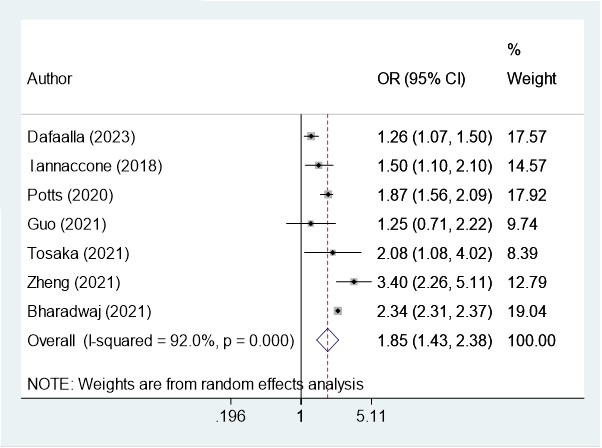

Supplement: S19 Fig — (JPG) [file pone.0318437.s019.jpg]

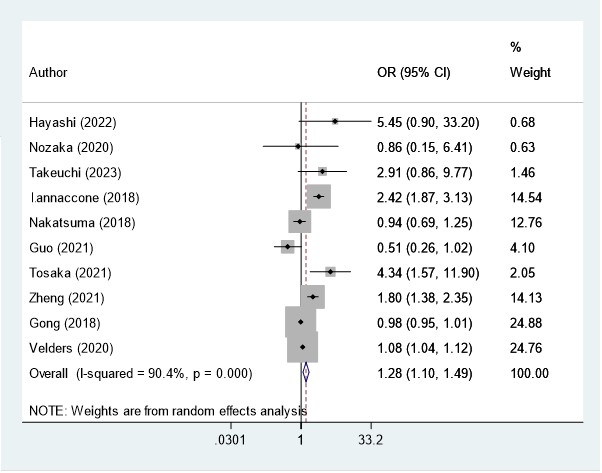

Supplement: S20 Fig — (JPG) [file pone.0318437.s020.jpg]

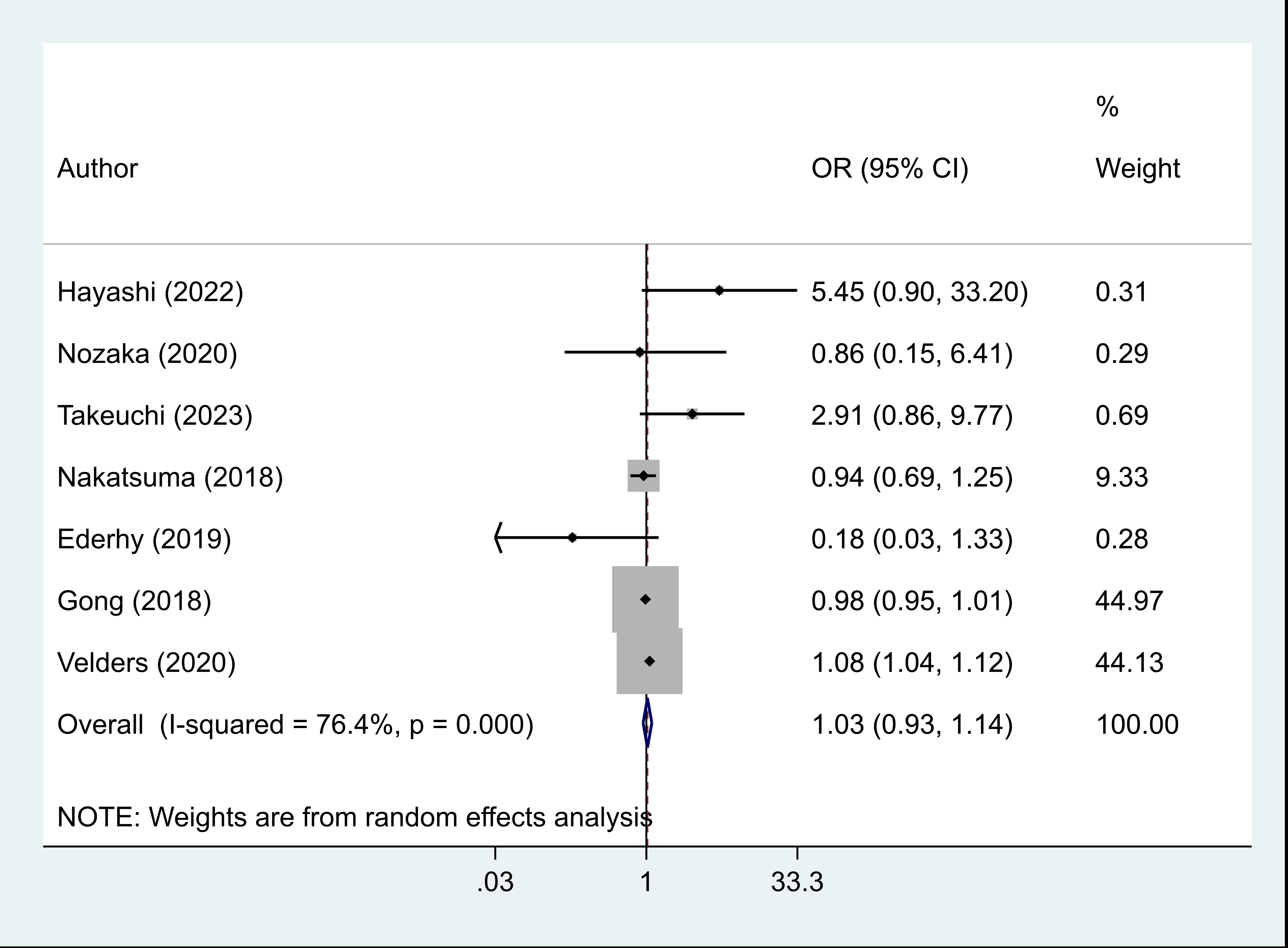

Supplement: S21 Fig — (JPG) [file pone.0318437.s021.jpg]

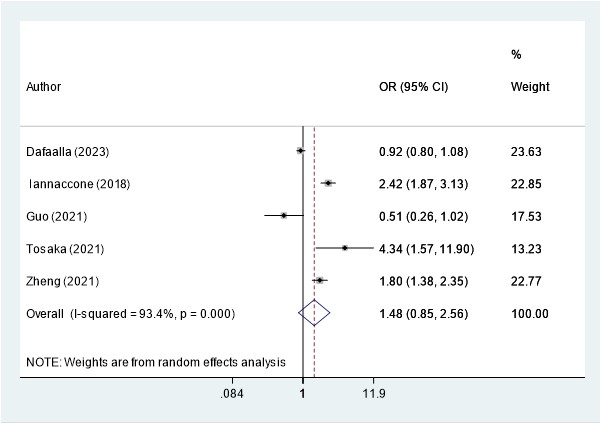

Supplement: S22 Fig — (JPG) [file pone.0318437.s022.jpg]

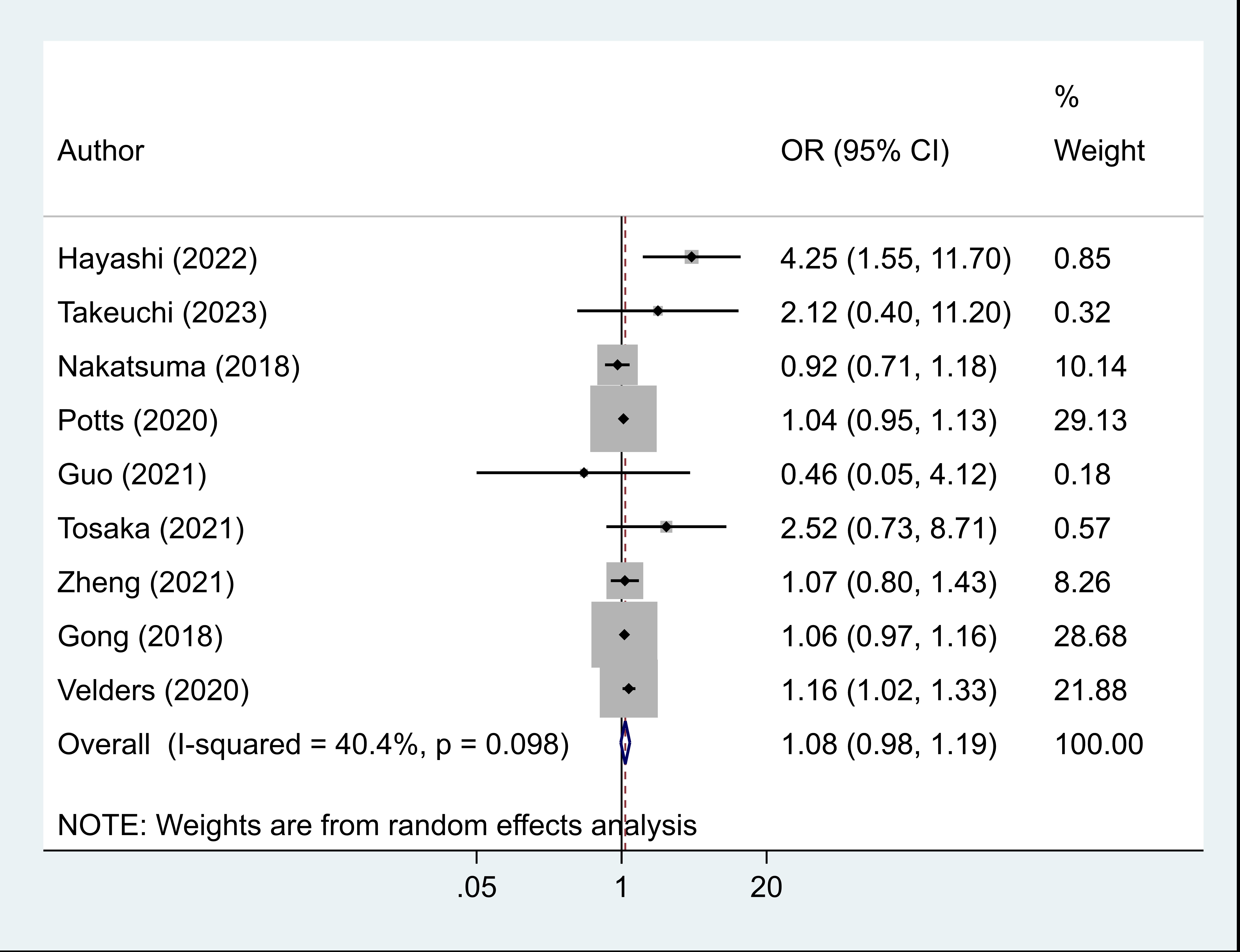

Supplement: S23 Fig — (JPG) [file pone.0318437.s023.jpg]

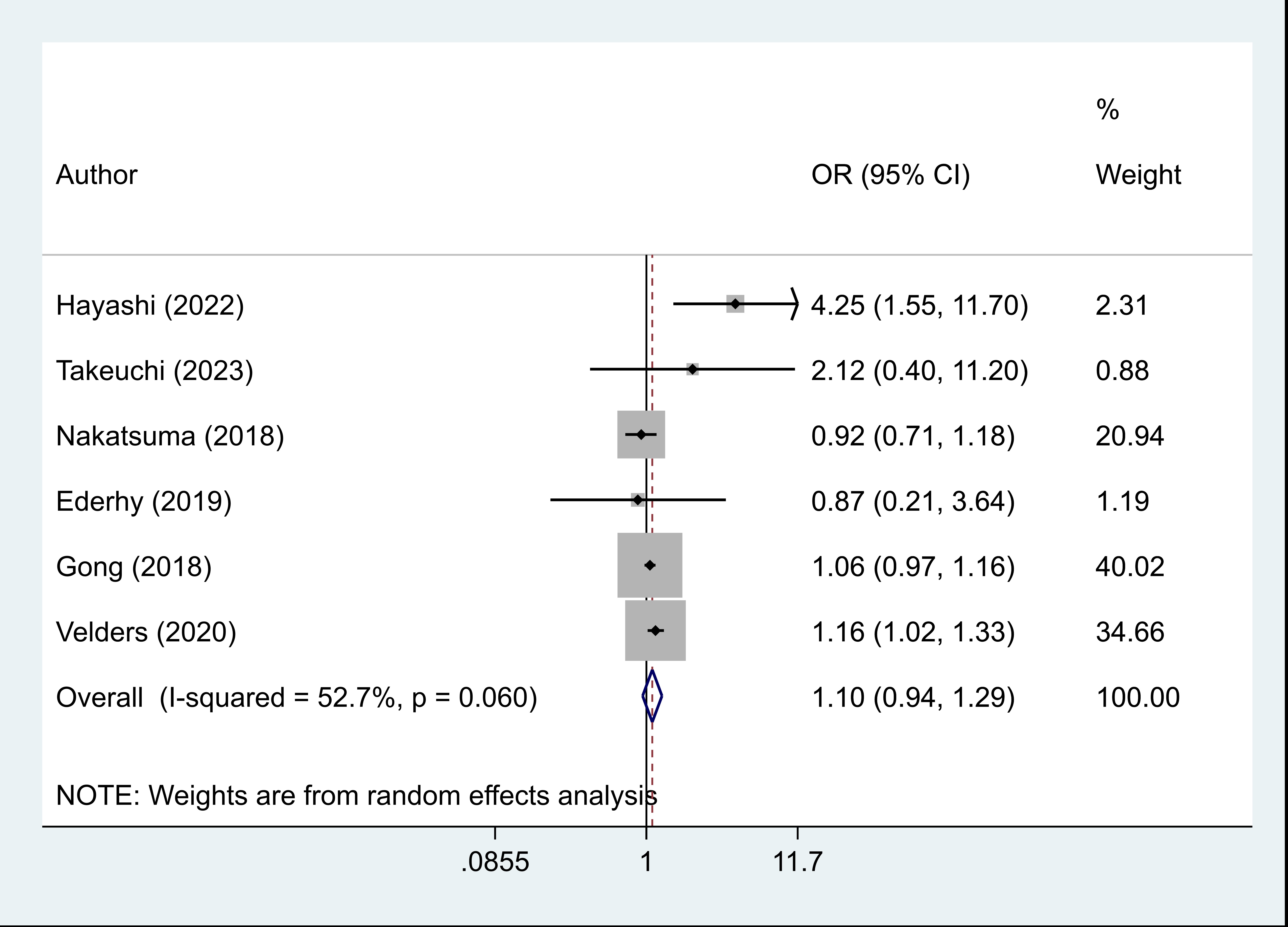

Supplement: S24 Fig — (JPG) [file pone.0318437.s024.jpg]

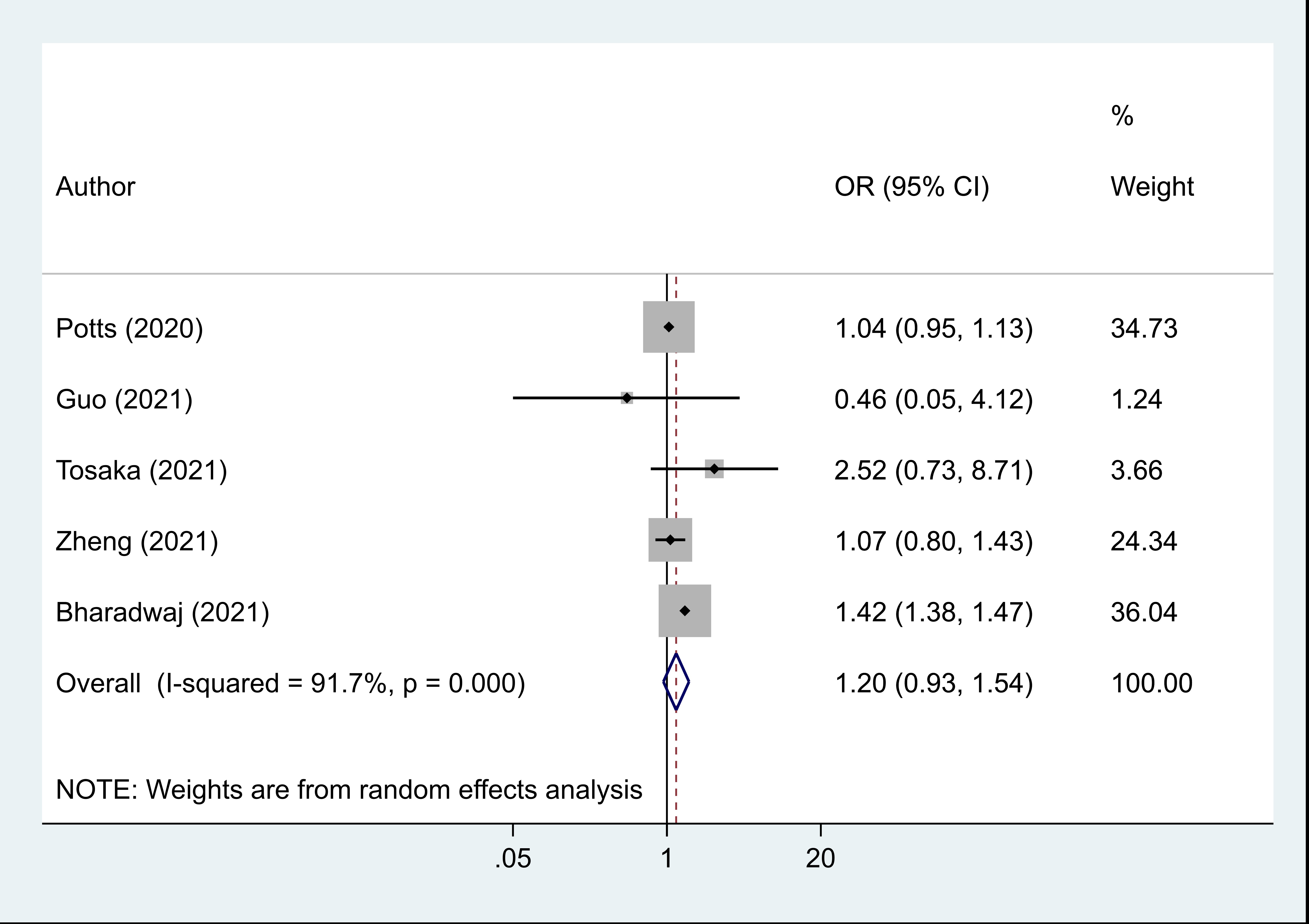

Supplement: S25 Fig — (JPG) [file pone.0318437.s025.jpg]
