# Supplementary material for: A meta-analysis on the impact of concurrent or pre-existing cancer diagnosis on acute myocardial infarction outcomes
Source: PLoS One. 2025 Jan 31;20(1):e0318437. doi: 10.1371/journal.pone.0318437 (PMC11785289; doi:10.1371/journal.pone.0318437)
Supplement: S1 Table — (DOCX) [file pone.0318437.s026.docx]

**S1 Table. Search strategy in PubMed**

("acute myocardial infarction"[MeSH Terms] OR "myocardial infarction"[Title/Abstract] OR "heart attack"[Title/Abstract] OR "acute coronary syndrome"[Title/Abstract]) AND ("neoplasms"[MeSH Terms] OR "cancer"[Title/Abstract] OR "malignancy"[Title/Abstract] OR "tumor"[Title/Abstract] OR "oncology"[Title/Abstract] OR "pre-existing cancer"[Title/Abstract] OR "concurrent cancer"[Title/Abstract]) AND ("mortality"[MeSH Terms] OR "survival"[Title/Abstract] OR "hospitalization"[Title/Abstract] OR "complications"[Title/Abstract] OR "outcomes"[Title/Abstract] OR "major adverse cardiovascular events"[Title/Abstract] OR "MACE"[Title/Abstract])
