# Supplementary material for: A meta-analysis on the impact of concurrent or pre-existing cancer diagnosis on acute myocardial infarction outcomes
Source: PLoS One. 2025 Jan 31;20(1):e0318437. doi: 10.1371/journal.pone.0318437 (PMC11785289; doi:10.1371/journal.pone.0318437)
Supplement: S2 Table — (DOCX) [file pone.0318437.s027.docx]

**S2 Table. Search strategy in EMBASE**

('acute myocardial infarction'/exp OR 'myocardial infarction':ti,ab OR 'heart attack':ti,ab OR 'acute coronary syndrome':ti,ab) AND ('neoplasm'/exp OR 'cancer':ti,ab OR 'malignancy':ti,ab OR 'tumor':ti,ab OR 'oncology':ti,ab OR 'pre-existing cancer':ti,ab OR 'concurrent cancer':ti,ab) AND ('mortality'/exp OR 'survival':ti,ab OR 'hospitalization':ti,ab OR 'complication':ti,ab OR 'outcome':ti,ab OR 'major adverse cardiovascular event':ti,ab OR 'MACE':ti,ab) AND [english]/lim
