# Supplementary material for: A meta-analysis on the impact of concurrent or pre-existing cancer diagnosis on acute myocardial infarction outcomes
Source: PLoS One. 2025 Jan 31;20(1):e0318437. doi: 10.1371/journal.pone.0318437 (PMC11785289; doi:10.1371/journal.pone.0318437)
Supplement: S3 Table — (DOCX) [file pone.0318437.s028.docx]

**S3 Table. Search strategy in Scopus**

(TITLE-ABS-KEY("acute myocardial infarction" OR "myocardial infarction" OR "heart attack" OR "acute coronary syndrome")) AND (TITLE-ABS-KEY("cancer" OR "neoplasm" OR "malignancy" OR "tumor" OR "oncology" OR "pre-existing cancer" OR "concurrent cancer")) AND (TITLE-ABS-KEY("mortality" OR "survival" OR "hospitalization" OR "complication" OR "outcome" OR "major adverse cardiovascular event" OR "MACE")) AND (LIMIT-TO(DOCTYPE, "ar")) AND (LIMIT-TO(LANGUAGE, "English")) AND (LIMIT-TO(SRCTYPE, "j"))
