# Supplementary material for: A meta-analysis on the impact of concurrent or pre-existing cancer diagnosis on acute myocardial infarction outcomes
Source: PLoS One. 2025 Jan 31;20(1):e0318437. doi: 10.1371/journal.pone.0318437 (PMC11785289; doi:10.1371/journal.pone.0318437)
Supplement: S4 Table — (DOCX) [file pone.0318437.s029.docx]

**S4 Table. Author’s judgements about study quality using the Newcastle Ottawa Risk of Bias Assessment tool**

|  | Dafaalla et al | Hayashi et al | Koo et al | Nozaka et al | Takeuchi et al | Ye_A et al | Ye_B et al | Velders et al | Wang et al | Landes et al | Iannaccone et al |
| --- | --- | --- | --- | --- | --- | --- | --- | --- | --- | --- | --- |
| Representativeness/appropriateness of participant selection  Random or consecutive recruitment=Y  Convenience sample=N  Not reported or unclear | Y | Y | Y | N | Y | Y | Y | Y | Y | Y | Y |
| Control for baseline differences in cohorts  Similarity of groups at baseline or adjustment in analyses=Y  No attempt to control or adjust=N  Not reported=NR | Y | Y | Y | Y | Y | Y | Y | Y | Y | Y | Y |
| Loss to follow-up  Explanation provided for loss of participants and/or intention to treat=Y  No explanation =N | Y | Y | N | Y | N | Y | Y | N | Y | Y | Y |
| Masking of exposure to outcomes assessor  Description of masking=Y  No masking or no description =N | Y | Y | Y | Y | Y | Y | Y | Y | Y | N | Y |
| Ascertainment of condition  Description of ascertainment/diagnostic criteria=Y  No description or patient self-report=N | Y | Y | Y | Y | Y | Y | Y | Y | Y | Y | N |
| Documentation of other treatment modalities  Documentation=Y  No documentation=N | Y | N | N | Y | N | Y | Y | N | Y | Y | Y |
| Extent to which valid outcomes are described  Adequate description of outcome=Y  Insufficient detail regarding outcome or follow-up time=N | Y | Y | Y | Y | Y | Y | Y | Y | Y | Y | Y |
| Prespecification of harms, mode of harms collection  Description of a list of harms assessed or monitoring=Y  No such description or passive harms collection=N  No adverse events reported=NA | Y | Y | Y | N | Y | N | N | Y | Y | Y | Y |
| Financial Conflict of interest (COI)  Funding source reported=Y  Funding source not reported=N | Y | Y | Y | Y | Y | Y | Y | Y | Y | Y | Y |
| Total score | 9 | 8 | 7 | 7 | 7 | 8 | 8 | 7 | 9 | 8 | 8 |
