# Supplementary material for: A meta-analysis on the impact of concurrent or pre-existing cancer diagnosis on acute myocardial infarction outcomes
Source: PLoS One. 2025 Jan 31;20(1):e0318437. doi: 10.1371/journal.pone.0318437 (PMC11785289; doi:10.1371/journal.pone.0318437)
Supplement: S6 Table — (DOCX) [file pone.0318437.s031.docx]

**S6 Table. Quality of the pooled evidence using the GRADE assessment**

|  | **Number of studies with design** | **Certainty of the evidence (GRADE)** | **Effect size  (95% CI); I^2^** |
| --- | --- | --- | --- |
|  |  |  |  |
| Risk of in-hospital mortality | N=13 (All cohort) | ⨁⨁◯◯ Low ^a^ | OR 1.44,  (1.20 to 1.73); 90.2% |
| Risk of mortality at 30-days follow up | N=7 (All cohort) | ⨁⨁◯◯ Low ^a^ | OR 1.47,  (1.24 to 1.74); 92.2% |
| Risk of mortality at one year follow up | N=3 (All cohort) | ⨁⨁◯◯ Low ^a^ | HR 2.67,  (1.73 to 4.11); 92.0% |
| Risk of mortality at more than one year follow up | N=15 (All cohort) | ⨁⨁◯◯ Low ^a^ | HR 1.87,  (1.65 to 2.11); 94.0% |
| Risk of in-hospital mortality related to cardiovascular complications | N=4 (All cohort) | ⨁⨁◯◯ Low ^a^ | OR 2.06,  (1.17 to 3.65); 55.4% |
| Risk of cardiovascular-related mortality after one year of follow-up | N=10 (All cohort) | ⨁⨁◯◯ Low ^a^ | HR 1.20,  (0.96 to 1.50); 74.4% |
| Risk of major bleeding | N=12 (All cohort) | ⨁◯◯◯ Very low ^b^ | OR 1.74  (1.40 to 2.16); 95.8% |
| Risk of reinfarction | N=12 (All cohort) | ⨁⨁◯◯ Low ^a^ | OR 1.20  (1.05, 1.37); 88.9% |
| Risk of stroke | N=11 (All cohort) | ⨁⨁◯◯ Low ^a^ | OR 1.16  (0.99, 1.37); 89.3% |

^a^Downgraded two-levels for non-randomized studies and for serious inconsistency (high heterogeneity)

^b^Downgraded three-levels for non-randomized studies, for serious inconsistency (high heterogeneity) and for presence of publication bias

OR: odds ration; HR: hazard ratio
