# Supplementary material for: A meta-analysis on the impact of concurrent or pre-existing cancer diagnosis on acute myocardial infarction outcomes
Source: PLoS One. 2025 Jan 31;20(1):e0318437. doi: 10.1371/journal.pone.0318437 (PMC11785289; doi:10.1371/journal.pone.0318437)
Supplement: S2 File — (DOC) [file pone.0318437.s033.doc]

**S2 File. Studies excluded after full text review**

Rohrmann S, Witassek F, Erne P, Rickli H, Radovanovic D. Treatment of patients with myocardial infarction depends on history of cancer. Eur Heart J Acute Cardiovasc Care. 2018 Oct;7(7):639-645. doi: 10.1177/2048872617729636. Epub 2017 Sep 19. PMID: 28927294.

Gon Y, Zha L, Sasaki T, Morishima T, Ohno Y, Mochizuki H, Sobue T, Miyashiro I. Heart Disease Mortality in Cancer Survivors: A Population-Based Study in Japan. J Am Heart Assoc. 2023 Dec 5;12(23):e029967. doi: 10.1161/JAHA.123.029967. Epub 2023 Nov 28. PMID: 38014664; PMCID: PMC10727325.

Paterson DI, Wiebe N, Cheung WY, Mackey JR, Pituskin E, Reiman A, Tonelli M. Incident Cardiovascular Disease Among Adults With Cancer: A Population-Based Cohort Study. JACC CardioOncol. 2022 Mar 15;4(1):85-94. doi: 10.1016/j.jaccao.2022.01.100. PMID: 35492824; PMCID: PMC9040097.

Tang M, Wang Y, Cao X, Day JD, Liu H, Sun C, Li G. In-hospital and one-year outcomes in cancer patients receiving percutaneous coronary intervention for acute myocardial infarction: A real-world study. Front Cardiovasc Med. 2023 Feb 7;9:1005473. doi: 10.3389/fcvm.2022.1005473. PMID: 36824290; PMCID: PMC9941959.

Willems RAL, Winckers K, Biesmans C, de Vos-Geelen J, Ten Cate H. Evolving data on cardiovascular complications in cancer. Thromb Res. 2022 May;213 Suppl 1:S87-S94. doi: 10.1016/j.thromres.2022.01.003. Epub 2022 May 26. PMID: 36210568.

Roule V, Verdier L, Blanchart K, Ardouin P, Lemaitre A, Bignon M, Sabatier R, Alexandre J, Beygui F. Systematic review and meta-analysis of the prognostic impact of cancer among patients with acute coronary syndrome and/or percutaneous coronary intervention. BMC Cardiovasc Disord. 2020 Jan 30;20(1):38. doi: 10.1186/s12872-020-01352-0. PMID: 32000685; PMCID: PMC6993442.

Galimzhanov A, Istanbuly S, Tun HN, Ozbay B, Alasnag M, Ky B, Lyon AR, Kayikcioglu M, Tenekecioglu E, Panagioti M, Kontopantelis E, Abdel-Qadir H, Mamas MA. Cardiovascular outcomes in breast cancer survivors: a systematic review and meta-analysis. Eur J Prev Cardiol. 2023 Dec 21;30(18):2018-2031. doi: 10.1093/eurjpc/zwad243. PMID: 37499186.
